# Mathematical modeling and analysis of insulin clearance *in vivo*

**DOI:** 10.1186/1752-0509-2-43

**Published:** 2008-05-13

**Authors:** Markus Koschorreck, Ernst Dieter Gilles

**Affiliations:** 1 Max Planck Institute for Dynamics of Complex Technical Systems, Sandtorstr. 1, 39106 Magdeburg, Germany

## Abstract

**Background:**

Analyzing the dynamics of insulin concentration in the blood is necessary for a comprehensive understanding of the effects of insulin *in vivo*. Insulin removal from the blood has been addressed in many studies. The results are highly variable with respect to insulin clearance and the relative contributions of hepatic and renal insulin degradation.

**Results:**

We present a dynamic mathematical model of insulin concentration in the blood and of insulin receptor activation in hepatocytes. The model describes renal and hepatic insulin degradation, pancreatic insulin secretion and nonspecific insulin binding in the liver. Hepatic insulin receptor activation by insulin binding, receptor internalization and autophosphorylation is explicitly included in the model. We present a detailed mathematical analysis of insulin degradation and insulin clearance. Stationary model analysis shows that degradation rates, relative contributions of the different tissues to total insulin degradation and insulin clearance highly depend on the insulin concentration.

**Conclusion:**

This study provides a detailed dynamic model of insulin concentration in the blood and of insulin receptor activation in hepatocytes. Experimental data sets from literature are used for the model validation. We show that essential dynamic and stationary characteristics of insulin degradation are nonlinear and depend on the actual insulin concentration.

## Background

Insulin regulates important physiological processes like cellular glucose uptake [1,2], metabolism [2,3] and gene expression [4]. The processes triggered by insulin are associated with widely spread diseases. Type I diabetes mellitus results from defective pancreatic insulin secretion [5,6]. Insulin resistance, obesity and type II diabetes mellitus may result from defects in the insulin signaling system [6-8] and are often accompanied by abnormalities in insulin degradation [9]. Improving therapies of these maladies is a topic of intense investigation [5,10,11].

### Insulin dynamics *in vivo*

A prerequisite for fully understanding the effects of insulin *in vivo *is to enlighten the fate of insulin after the injection or endogenous production. Much work has been done in past decades to study insulin kinetics in the blood [12-14]. In the last few years, efforts have been focused on analyzing the dynamics of insulin concentration after the subcutaneous injection [15-17]. The resulting models describe insulin removal from the blood in a highly reduced way [12,17], whereas the subcutaneous tissue is usually modeled in more detail. Insulin traverses different compartments (e.g. the injection pocket and the interstitium) after the injection and can be degraded or temporarily stored within these compartments [17].

Long acting insulins tend to form dimers or hexamers in the subcutaneous tissue, whereas fast acting insulin analogues have a decreased ability to form oligomers [5]. Oligomer formation slows down the transition of insulin from the injection pocket in the subcutaneous tissue to the blood. These effects are included in some models [17]. In other studies, insulin dynamics are linked with glucose dynamics [18-23]. The corresponding models describe all involved processes in a highly reduced way.

There are also efforts to predict glucose concentration and to automate insulin dosage for individuals with impaired glucose levels [24-29]. These efforts are first steps towards the development of an artificial pancreas [30].

In the last few decades, many different kinetics for insulin removal from the blood were proposed. The most frequently used kinetics are linear first order kinetics, Michaelis-Menten kinetics or a combination of both [13]. Due to the investigation of narrow concentration intervals, nonlinearity was difficult to demonstrate [31]. The presence of nonlinearities due to saturable processes now is widely accepted [5,9]. However, insulin degradation is described as a linear first order process in most models. Allocation of insulin degradation to specific tissues is not performed in the models of insulin dynamics [17]. Therefore, no model-based analysis of the contributions of the liver and the kidney to the degradation process has been done. A prerequisite for such an analysis is the availability of a validated model describing all important processes.

### Insulin receptor dynamics *in vitro*

There are several models in literature that describe insulin receptor dynamics *in vitro*. Most models [32-36] focus on a subset of the occurring processes and lump several processes into single reaction steps. This reduces the number of model parameters and has to be done if there is only little experimental data and if there are many parameters to estimate. However, two recent *in vitro *models describe insulin receptor dynamics in more detail [37,38].

Sedaghat et al. combined models of insulin binding [36] and receptor internalization, recycling and degradation [35] and extended them to a mathematical model of insulin signaling in adipocytes [37]. Model parameters were taken from other models and *in vitro *experiments. The receptor part of this model includes the binding of two insulin molecules as well as phosphorylation, internalization, degradation and synthesis of the receptor. A very strong coupling between insulin binding and receptor phosphorylation is assumed. The second insulin molecule can only bind to the receptor if the receptor is phosphorylated. Dephosphorylation of the receptor (with simultaneous insulin dissociation) is only possible if only one insulin molecule is bound to the receptor. Phosphorylated receptors without insulin are not part of the model.

Hori et al. described receptor phosphorylation, internalization and recycling in Fao hepatoma cells [38] at 100 *nM *insulin. They analyzed several models corresponding to different model assumptions and different levels of detail. Model parameters were estimated using experimental data sets from literature. The main limitations of the models of Hori et al. [38] are that they are only valid at 100 *nM *insulin and that insulin binding is not explicitly included. Due to the high insulin concentration (100 *nM*), all receptors at the plasma membrane are assumed to be liganded. Hori et al. also provide a general model structure without parameterization that includes the binding of one insulin molecule to the receptor and is intended for variable insulin concentrations. As above, receptor dephosphorylation and insulin dissociation are coupled in all models. Insulin dissociation is a prerequisite for receptor dephosphorylation or the processes are lumped into a single step. In addition, most processes are assumed to be irreversible.

Thus, there are many couplings between different processes in all detailed receptor models [37,38].

### Insulin dynamics and insulin receptor dynamics *in vivo*

*In vivo *models from literature predict insulin or glucose concentrations in the blood after the injection of glucose or insulin. The physiological state of the involved insulin-responsive tissues, e.g. the activation of insulin receptors, cannot be obtained from these *in vivo *models, as their level of detail is quite low [12-17]. Though insulin dynamics in the blood and insulin receptor activation are highly related, no detailed analysis of the interactions between these processes exists in literature. Hovorka et al. [14] took a first step in this direction. However, the receptor part of their model only distinguishes between free receptors and receptors with bound insulin. In addition, the focus of this study is clearly on insulin kinetics.

*In vivo *models describing hepatic processes in such a detailed way as *in vitro *models [37,38] could be of great interest for a deeper understanding and a model based control of insulin and glucose dynamics. Additionally, a detailed model could serve as a starting point for modeling and analysis of the signaling cascades emerging from the hepatic insulin receptor *in vivo*. Due to combinatorial complexity in signal transduction [39], reduced order modeling techniques [40,41] will have to be used to describe insulin signaling comprehensively.

We present a literature-based mathematical model of insulin dynamics and hepatic insulin receptor activation in rats. Compared to other models [32-38], we describe receptor processes in more detail to get insights into the processes and into the connections between insulin dynamics and insulin receptor activation in hepatocytes. This enables us to simultaneously analyze insulin dynamics in the blood and insulin receptor dynamics in the liver. In contrast to other studies, we decouple insulin binding and dissociation from receptor phosphorylation, as there is experimental evidence that receptor phosphorylation does not affect insulin binding [42]. In addition, we model receptor phosphorylation as a reversible process if insulin is bound to the receptor. We take experimentally determined *in vitro *parameters for each reaction, wherever this is possible. The result is a physiologically well founded mechanistic model that does not couple or lump different processes. Almost all processes were parameterized by values from literature. The remaining parameter values could be derived from physiological considerations.

Model validation is performed with experimental data sets from literature. We emphasize that the data sets used for the model validation are not used for parameter estimation. This corresponds to a strict separation of model construction and model validation which is frequently applied [43]. A very remarkable result of the model validation is that the model with parameters from literature is able to match experimental data sets.

We perform a detailed stationary analysis of the contributions of the liver and the kidney to insulin degradation and insulin clearance as well as of the activation state of hepatic insulin receptors under varying insulin concentrations.

## Results and Discussion

### The model

The model consists of ordinary differential equations (ODEs) and describes the dynamic behavior of radioactively labeled and unlabeled insulin in the blood and the physiological state of hepatic insulin receptors. It can also be used for the injection of only labeled or only unlabeled insulin. Distinction between labeled and unlabeled insulin is necessary as unlabeled insulin is synthesized in the pancreas whereas labeled insulin is not. Therefore, in experiments with labeled insulin, the fraction of labeled insulin changes over time.

Almost all state variables in the model represent concentrations and are given in *nM*. Exceptions are the state variables *Ins*_*ub *_and Ins_*,*ub *_that represent amounts of substances and are given in *nmol*. All rates are given in *nM*·*s*^-1^. The rates describing insulin receptor dynamics (*r*_*j*_, *i*_*j *_and *f*_*j*_, *j ε *ℕ) refer to the hepatocyte volume *v*_*hep*_. All other rates refer to blood plasma volume *v*_*p*_. The executable model is given in MATLAB format in Additional file 1. We also provide the receptor part of the model as an independent model that can be used for the simulation of *in vitro *experiments (Additional file 2).

#### Important tissues and processes

The liver and the kidney are the most important insulin degrading tissues [5,9]. However, fat and muscle tissues also contribute to insulin degradation. In the following, we show that the insulin degradation rate of the fat tissue is small compared to the hepatic insulin degradation rate. According to Sedaghat et al. [37], the total receptor concentration in adipocytes is 10^-3 ^*nM *and the rate constant of receptor internalization is 3.5·10^-5 ^*s*^-1^. Insulin receptors in hepatocytes have a minimal internalization rate constant of 2·10^-4 ^*s*^-1 ^[34] and a concentration of 40 *nM *(10^5 ^receptors per hepatocyte [44], a hepatocyte is assumed to be a sphere with 20 *μm *diameter). 78% of the liver volume is occupied by hepatocytes [45]. Liver mass is about 5% of body weight [46], the mass of the fat tissue is in the same order of magnitude. We postulate the same insulin binding characteristics to the receptor in both tissues. The product of receptor concentration and the kinetic parameter for receptor internalization in adipocytes is five orders of magnitude lower than in hepatocytes. Therefore, the contribution of the fat tissue to insulin degradation can be neglected. The contribution of the muscle tissue will not be analyzed either. No quantitative data was found and a qualitatively similar behavior in comparison to the liver is expected.

Hepatic insulin receptors have access to insulin molecules in the space of Disse [46]. The space of Disse (perisinusoidal space) contains blood plasma and is an extracellular space between liver sinusoids (special blood vessels) and hepatocytes. Insulin molecules from the space of Disse can be bound and are internalized together with the receptor. In the space of Disse, there is also nonspecific insulin binding to hepatocytes. Inside the cell, in the acidic endosomal compartment, insulin dissociates from the receptor and is degraded. The receptor then recycles to the cell surface [1].

Our model explicitly describes dynamic insulin receptor activation in hepatocytes of the liver. Processes considered are insulin binding to the receptor, receptor autophosphorylation, internalization and recycling. Compared to other models of the insulin receptor which also include these processes [37,38], we provide an extended description, model the *in vivo *situation and include reversible nonspecific insulin binding.

The kidney's contribution to insulin clearance mainly consists of the filtering of insulin from the blood [9]. The filtering function of the kidney is modeled as a degradation rate that, according to experimental data [47], does not saturate and is proportional to insulin concentration in the plasma.

Pancreatic insulin secretion is mainly induced by plasma glucose [2]. As we focus on insulin degradation, glucose is not included in the model. We model pancreatic insulin secretion in a highly simplified way as a function of insulin concentration. Due to high robustness to changes in the parameters for insulin secretion (Additional file 3), this simplification does not lead to significant approximation errors. In addition, insulin secretion is irrelevant for stationary analysis at constant insulin concentrations.

Altogether, our model describes the following processes: intravenous injection of radioactively labeled and unlabeled insulin, pancreatic insulin secretion, hepatic and renal insulin degradation, hepatic insulin receptor activation and nonspecific insulin binding by the liver.

#### Parameterization of the model

*In vivo *model parameters cannot be measured directly in most cases. Taking parameters from *in vitro *experiments or models for *in vivo *processes is a promising alternative. Note that this can be problematic since the *in vitro *parameter values may not be similar to their *in vivo *counterparts. However, it is the only possibility if there is not sufficient experimental data and a model structure that guarantees identifiability. Using *in vitro *parameters or otherwise determined model parameters and linking kinetic models of smaller parts of the overall system together is frequently performed, e.g. by the Silicon Cell project [43]. This strategy is structurally supported by modular modeling tools, e.g. ProMoT [48].

In this study, model parameters (Table 1) are taken from previously published *in vitro *experiments [47,49-55] and small models of insulin binding [36], receptor internalization [34] and nonspecific hepatic insulin binding [46]. The models from literature [34,36,46] were combined and kinetic parameters for the remaining processes were taken from *in vitro *data.

**Table 1:** Model parameters and initial conditions

Parameter	Value	Source	Meaning of the parameter
*kins*	10^-3 ^*nM*^-1 ^*s*^-1^	[36]	insulin binding to the receptor
*kins*1*d*	4·10^-4 ^*s*^-1^	[36]	insulin dissociation from the receptor (I1, PM)
*kins*2*d*	4·10^-2 ^*s*^-1^	[36]	insulin dissociation from the receptor (I2, PM)
*kins*1*den*	1.925·10^-3 ^*s*^-1^	[49]	insulin dissociation from the receptor (I1, EN)
*kins*2*den*	3.85·10^-3 ^*s*^-1^	[50]	insulin dissociation from the receptor (I2, EN)
*kyd*	3.85·10^-3 ^*s*^-1^	[51]	receptor dephosphorylation (PM)
*kyden*	7.22·10^-3 ^*s*^-1^	[52]	receptor dephosphorylation (EN)
*kyp*	0.0231 *s*^-1^	[52]	autophosphorylation of the receptor (I1 and I2)
*intk*1	5.5·10^-4 ^*s*^-1^	[34]	internalization of phosphorylated receptors
*intk*2	2·10^-4 ^*s*^-1^	[34]	internalization of unphosphorylated receptors
*reck*1	1.533·10^-3 ^*s*^-1^	[34]	recycling of receptors without insulin
*k*1*ub*	0.35 *s*^-1^	[46]	nonspecific insulin binding in the liver
*k*2*ub*	0.2 *s*^-1^	[46]	dissociation of nonspecifically bound insulin
*pansec*	0.0016976 *nM*·*s*^-1^	calc.	pancreatic insulin secretion
*K pan*	0.5 *nM*	ass.	concentration of half-maximal insulin secretion
*m*_*liver*_	0.05·*m*_*body*_	[46]	mass of the liver
*v*_*p*_	0.03375·10^-3 ^*l*·*g*^-1^·*m*_*body*_	[54]	plasma volume
*ρ*_*liver*_	1.051·10^3 ^g·*l*^-1^	[53]	density of the liver
*v*_*hep*_	(*m*_*liver*_/*ρ*_*liver*_)·0.78	[45]	total hepatocyte volume
*v*_*d*_	0.272·10^-3 ^*l*·*g*^-1^·*v*_*hep*_·*ρ*_*liver*_	[46]	volume of the space of Disse
*m*_*kidney*_	2·0.85 *g*·*m*_*body*/_(230 *g*)	[55]	mass of the kidney
*K kidney*	0.0225·10^-3 ^*l*·(*s*·*g*)^-1^·*m*_*kidney*_	[47]	clearance of the kidney

Note that *m*_*body *_(body weight in *g*), *t*_*in *_(injection time in *s*), *n*_*in *_and n_*,*in *_(amounts of injected unlabeled and labeled insulin in *nmol*) are also model parameters. We do not give values for them in this table as they depend on the analyzed scenario. Note that the model can be used for rats of arbitrary body weights as well as for different injection times and amounts of injected labeled and unlabeled insulin. Initial conditions are: *Ins *= 0.07, Ins* = 0, *R *= 31.619, *IR *= 0.430007, *I*2*R *= 0.000696311, *Rp *= 0.227528, *IRp *= 2.07275, *I*2*Rp *= 0.00363012, *R*_*en *_= 4.88528, *IR*_*en *_= 0.145537, *I*2*R*_*en *_= 0.000121295, *Rp*_*en *_= 0.122602, *IRp*_*en *_= 0.492464, *I*2*Rp*_*en *_= 0.000433466, *Ins*_*ub *_= 1.29948·10^-6^·*m*_*body*_, Ins_*,*ub *_= 0. The unit of *Ins*_*ub *_and Ins_*,*ub *_is *nmol*, the unit of all other state variables is *nM*. ass.: assumption, calc.: calculation (see Additional file 4), EN: in endosomes, PM: at the plasma membrane, *I*1: one insulin molecule bound to the receptor, *I*2: two insulin molecules bound to the receptor.

The parameters from the models of insulin binding [36] and nonspecific insulin binding in the liver [46] were directly taken for our model. The relatively simple models for receptor internalization and recycling at high insulin concentrations and without insulin [34] were combined to describe receptor internalization and recycling at arbitrary insulin concentrations.

The parameters for pancreatic insulin secretion were chosen to guarantee the physiological basal level of insulin (0.07 *nM*, Gisela Drews, personal communication) and to cut off insulin synthesis at peak concentrations in insulin therapy (0.5 *nM *[16,17]).

This study uses the rat as model organism because much more parameters are known for rats than for humans. The model validation is performed using experimental data sets for rats.

All volumes are assumed to be constant. In addition, all tissues are assumed to contact the same total insulin concentration, which is the sum of labeled and unlabeled insulin. The physiological justification of this assumption is the high heart rate of rats (320 – 480 *bpm *[54]) that guarantees a fast distribution of circulating insulin.

#### The liver

**Figure 1:**
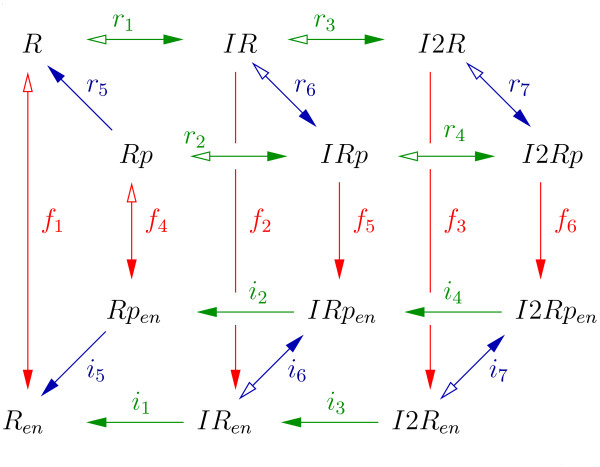
**Insulin receptor activation in hepatocytes**. The receptor is denoted as *R*. One or two insulin molecules can bind to the receptor (green arrows). This is indicated by a prefix *I *or *I*2, respectively. Receptor phosphorylation (blue arrows) is indicated by a suffix *p*, receptor internalization to the endosomal compartment (red arrows) is indicated by a subscript *en*. Arrows with two heads indicate reversible reactions. Arrows with one head indicate irreversible reactions. Filled arrowheads indicate positive direction of rates.

Insulin degradation in hepatocytes is modeled in a very detailed way. The described processes are successive binding of two insulin molecules to the insulin receptor, receptor phosphorylation and receptor internalization (Figure 1). In accordance with experimental results [56], the described processes lead to saturation of hepatic insulin degradation at high insulin concentrations.

Model assumptions that are supported by studies from literature are:

• *Insulin binding and dissociation are independent of the phosphorylation state of the receptor*. This is directly supported by experimental evidence [42].

• *Only receptors with bound insulin show autophosphorylation activity*. Autophosphorylation is induced by insulin binding [2] and there is no experimental data quantifying autophosphorylation of receptors without bound insulin.

• *Receptor dephosphorylation is independent of insulin binding*. Receptor dephosphorylation is performed by protein phosphatases [2]. It seems very unlikely that insulin binding to the extracellular *α*-chain of the receptor induces conformational changes in the intracellular *β*-chain that are large enough to significantly change the affinity of phosphatases for their phosphorylated substrate sites.

• *Insulin dissociation from endosomal receptors is irreversible*. Upon internalization, the pH in endosomes decreases rapidly, which promotes insulin dissociation from the receptor [9]. Free endosomal insulin is degraded by proteases [9].

• *Only receptors without insulin are recycled*. Receptor recycling is faster if there is no external insulin [34]. This leads to the assumption that an additional step for receptors with bound insulin is necessary before recycling is possible. A very promising candidate for this step is insulin dissociation from the receptor. In this case, a single rate constant for recycling, independent of insulin concentration is sufficient to explain the observation.

• *Phosphorylated receptors are internalized faster than unphosphorylated receptors*. In the presence of higher insulin concentrations, more insulin receptors are phosphorylated [2]. Receptor internalization is faster at high insulin concentrations than without external insulin [34]. In addition, there are reports that receptor internalization depends on phosphorylation [9].

• *Labeled and unlabeled insulin show the same physiological characteristics*. Labeling of the insulin molecules was performed with ^125^I [57-59]. The size of this modification is small compared to the size of the insulin molecule and should not change its binding characteristics, the effect on receptor phosphorylation, the rate of nonspecific insulin binding or the rate of renal insulin filtration.

• *All processes in hepatocytes obey mass action kinetics*. The processes that were adopted from other models obey mass action kinetics [34,36,46]. Mass action kinetics is a good and frequently used approximation for processes at the molecular level.

For the following assumptions there is no experimental data in literature supporting them. These assumptions were made to keep the number of parameters as low as possible.

• *Receptors with one or two bound insulin molecules show the same autophosphorylation activity*.

• *Receptor recycling is independent of receptor phosphorylation*.

In the following, the insulin receptor is denoted as *R*. The binding of one or two insulin molecules is indicated by a prefix *I *or *I*2, respectively. A suffix *p *indicates receptor phosphorylation, a subscript *en *indicates internalization to the endosomal compartment. All concentrations of receptor species refer to *v*_*hep*_, the total volume of hepatocytes.

In general, rates denoted by the standard notation *r*_*j *_describe processes at the plasma membrane of hepatocytes or outside the hepatocytes (nonspecific insulin binding, pancreatic insulin secretion and renal insulin removal). Rates denoted by *i*_*j *_describe *internal *processes occurring in endosomes of hepatocytes, and rates denoted by *f*_*j *_describe *flows *between the plasma membrane and endosomes of hepatocytes.

Figure 1 shows the reaction scheme of processes in hepatocytes.

The hepatocyte part of the model does not distinguish between labeled and unlabeled insulin, which reduces the number of necessary ODEs. Hepatocytes have contact to the total insulin concentration *Ins *that is the sum of labeled and unlabeled insulin concentrations. The concentration of labeled insulin is denoted as Ins_* _. Unlabeled insulin (*Ins *– Ins_*_) has no separate notation. The total contribution of the liver to insulin degradation is

*r*_*liv *_= (-*r*_1 _- *r*_2 _- *r*_3 _- *r*_4_)·*v*_*hep*_/*v*_*p*_.

The plasma volume is denoted as *v*_*p*_, the total hepatocyte volume is denoted as *v*_*hep*_. Strictly speaking, *r*_*liv *_defines insulin removal from the blood, whereas insulin degradation is performed in hepatic endosomes. However, *r*_*liv *_is the contribution of the liver to insulin dynamics. In the stationary case, the values of the rates for insulin removal and insulin degradation are identical.

Rates *r*_1 _– *r*_4 _describe insulin binding to the insulin receptor at the plasma membrane. The values of the parameters *kins*, *kins*1*d *and *kins*2*d *were directly taken from the model of Wanant et al. [36].

r1=kins⋅R⋅Ins−kins1d⋅IRr2=kins⋅Rp⋅Ins−kins1d⋅IRpr3=kins⋅IR⋅Ins−kins2d⋅I2Rr4=kins⋅IRp⋅Ins−kins2d⋅I2Rp

Rates *r*_5 _– *r*_7 _describe receptor phosphorylation at the plasma membrane.

r5=kyd⋅Rpr6=kyp⋅IR−kyd⋅IRpr7=kyp⋅I2R−kyd⋅I2Rp

Rates *i*_1 _– *i*_4 _describe insulin dissociation from the receptor in endosomes.

i1=kins1den⋅IReni2=kins1den⋅IRpeni3=kins2den⋅I2Reni4=kins2den⋅I2Rpen

Rates *i*_5 _– *i*_7 _describe receptor phosphorylation in endosomes.

i5=kyden⋅Rpeni6=kyp⋅IRen−kyden⋅IRpeni7=kyp⋅I2Ren−kyden⋅I2Rpen

According to our model assumptions, unphosphorylated receptors without insulin (*R *and *R*_*en*_) have no autophosphorylation activity. Therefore, the reactions represented by the rates *r*_5 _and *i*_5 _are irreversible. Rates *f*_1 _– *f*_6 _describe receptor internalization and recycling.

f1=intk2⋅R−reck1⋅Renf2=intk2⋅IRf3=intk2⋅I2Rf4=intk1⋅Rp−reck1⋅Rpenf5=intk1⋅IRpf6=intk1⋅I2Rp

The value of the parameter *intk*1 was directly taken from a model of receptor internalization and recycling at high insulin concentrations [34]. The values of the parameters *intk*2 and *reck*1 are from a model of receptor internalization and recycling without insulin [34].

Altogether, the described processes result in the following balance equations for hepatic insulin receptor species.

$$\begin{array}{c} \dot{R}=-r_{1}+r_{5}-f_{1}\\ \dot{IR}=r_{1}-r_{3}-r_{6}-f_{2}\\ \dot{I2R}=r_{3}-r_{7}-f_{3}\\ \dot{R}p=-r_{2}-r_{5}-f_{4}\\ I\dot{R}p=r_{2}-r_{4}+r_{6}-f_{5}\\ \dot{I2Rp}=r_{4}+r_{7}-f_{6}\\ {\dot{R}}_{en}=i_{1}+i_{5}+f_{1}\\ \dot{IR_{en}}=-i_{1}+i_{3}-i_{6}+f_{2}\\ \dot{I2R_{en}}=-i_{3}-i_{7}+f_{3}\\ \dot{R}p_{en}=i_{2}-i_{5}+f_{4}\\ I\dot{R}p_{en}=-i_{2}+i_{4}+i_{6}+f_{5}\\ \dot{I2Rp_{en}}=-i_{4}+i_{7}+f_{6} \end{array}$$

The liver also performs nonspecific insulin binding. This reversible process does not saturate [46] and dampens rapid variations in insulin concentration. The rates *r*_*ub *_and r_*,*ub *_define nonspecific binding of unlabeled and labeled insulin, respectively.

$$\begin{array}{c} r_{ub}=(k1ub\cdot (\overset{\text{unlabeled insulin}}{\overset{︷}{Ins-Ins_{*}}})\cdot v_{d}-k2ub\cdot Ins_{ub})/v_{p}\\ r_{*,ub}=(k1ub\cdot Ins_{*}\cdot v_{d}-k2ub\cdot Ins_{*,ub})/v_{p} \end{array}$$

The values of the parameters *k*1*ub *and *k*2*ub *were directly taken from the model of Hammond et al. [46]. The volume of the space of Disse, in which nonspecific insulin binding takes place, is denoted as *v*_*d*_. The concentration of unlabeled insulin is *Ins *– Ins_* _(unit: *nM*), while Ins_*,*ub *_and *Ins*_*ub *_are the amounts of substance (unit: *nmol*) for nonspecifically bound labeled and unlabeled insulin, respectively. The expressions for the forward reactions of the rates *r*_*ub *_and r_*,*ub *_are multiplied by *v*_*d *_(unit: *l*) as *Ins *and Ins_* _(unit: *nM*) are concentrations, whereas *Ins*_*ub *_and Ins_*,*ub *_are amounts of substance (unit: *nmol*). The balances of the amounts of nonspecifically bound labeled and unlabeled insulin are given by

$$\begin{array}{c} I\dot{n}s_{ub}=r_{ub}\cdot v_{p}\\ I\dot{n}s_{*,ub}=r_{*,ub}\cdot v_{p}. \end{array}$$

In order to obtain the unit *nM*·*s*^-1 ^for all rates, we divide by *v*_*p *_within the rates *r*_*ub *_and r_*,*ub *_and multiply the rates by *v*_*p *_in the ODEs for *Ins*_*ub *_and Ins_*,*ub*_, emphasizing the need for *v*_*p*_.

Note that symbols with an asterisk indicate radioactively labeled insulin species. Species with insulin whose symbols do not contain an asterisk can contain labeled or unlabeled insulin, except for *Ins*_*ub*_, which only represents unlabeled nonspecifically bound insulin.

#### The kidney

The kidney performs insulin degradation by filtering insulin from the blood [5]. The degradation rate *r*_*kid *_is proportional to insulin concentration [47].

*r*_*kid *_= -*K kidney*·*Ins/v*_*p*_

Insulin clearance is defined as the quotient of the degradation rate and the insulin concentration [13]. Therefore, *K kidney *is the clearance of the kidney.

There are also reports that receptor-mediated transport in man contributes about one third to total renal insulin removal [9]. This may result in a slightly nonlinear behavior of renal insulin degradation. However, the nonlinearity resulting from receptor saturation is not visible in the experimentally examined concentration interval [47]. Therefore, linear first order kinetics are a good approximation of renal insulin degradation.

#### Insulin injection and secretion

Pancreatic insulin secretion is induced by plasma glucose [2], which is not included in the model. In the model, pancreatic insulin secretion *r*_*pan *_is modeled as a function of insulin concentration and turned off at high insulin concentrations. This corresponds to the implicit assumption that glucose dynamics are faster than insulin dynamics. Peak concentrations in insulin therapy are about 60 – 80 *μU*·*ml*^-1 ^[16,17], which is about 0.35 – 0.5 *nM*. Insulin secretion is assumed to be cut off smoothly at *K pan *= 0.5 *nM *with Hill coefficient 10. The physiological basal insulin concentration (0.07 *nM*) is guaranteed by adjusting the parameter *pansec *such that the secretion rate equals the sum of stationary insulin degradation rates of the liver and the kidney at 0.07 *nM *insulin (Additional files 4 and 5).

$$r_{pan}=pansec\cdot \left(1-\frac{Ins^{10}}{Ins^{10}+Kpan^{10}}\right)$$

Intravenous injection of labeled and unlabeled insulin (u_*,*in *_and *u*_*in*_) is performed during injection time *t*_*in *_with a constant injection rate that is sharply, but smoothly cut off with Hill coefficient 50. This is an arbitrarily chosen parameter that realizes a switching procedure. Real step functions may cause numerical problems which can be avoided in this way.

The amounts of injected unlabeled and labeled insulin are *n*_*in *_and n_*,*in *_(unit: *nmol*), respectively. Each parameter can be set to zero if no injection of labeled or unlabeled insulin is desired. The corresponding input functions *u*_*in *_or u_*,*in *_then equal zero.

$$\begin{array}{c} u_{in}=\frac{n_{in}}{t_{in}}\cdot \left(1-\frac{t^{50}}{t^{50}+t_{in}^{50}}\right)/v_{p}\\ u_{*,in}=\frac{n_{*,in}}{t_{in}}\cdot \left(1-\frac{t^{50}}{t^{50}+t_{in}^{50}}\right)/v_{p} \end{array}$$

Note that *u*_*in *_and u_*,*in *_are not defined for *t*_*in *_= 0 *s *which corresponds to a very rapid bolus injection of insulin. If *t*_*in *_= 0 *s *is nevertheless desired, this infinitely small injection time can be realized by setting the initial conditions directly to the corresponding values. The rates *u*_*in *_and u_*,*in *_then have to be set to zero.

$$\begin{array}{c} Ins=0.07+\frac{n_{in}+n_{*,in}}{v_{p}}\\ Ins^{*}=\frac{n_{*,in}}{v_{p}} \end{array}$$

Note that 0.07 *nM *is the basal concentration of insulin.

#### Insulin concentration in plasma

The balances of the concentrations of labeled (Ins_*_) and total insulin (*Ins*) are given by:

$$\begin{array}{c} \dot{Ins}=r_{liv}+r_{kid}+r_{pan}+u_{in}+u_{*,in}-r_{*,ub}-r_{ub}\\ {\dot{Ins}}_{*}=(r_{liv}+r_{kid})\cdot \frac{Ins_{*}}{Ins}+u_{*,in}-r_{*,ub}. \end{array}$$

Note that *r*_*liv *_and *r*_*kid *_refer to total insulin. Therefore, only their fractions Ins_*_/Ins that correspond to labeled insulin have to be considered in the balance of Ins_*_.

### Dynamic model validation

#### Insulin dynamics

**Figure 2:**
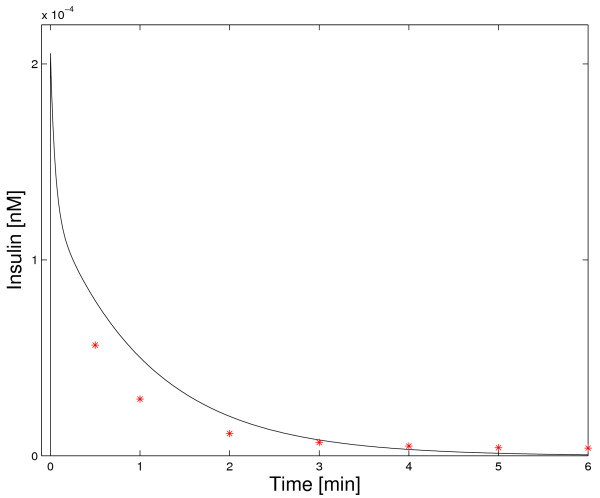
**Dynamic model validation: physiological insulin concentrations**. Simulation of the concentration of radioactively labeled insulin in plasma after the injection of a very low amount of radioactively labeled insulin is shown and compared to experimental data [59].

**Figure 3:**
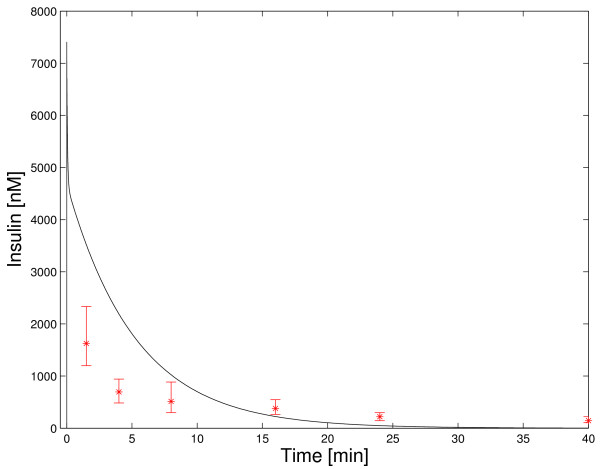
**Dynamic model validation: extremely high insulin concentrations**. Simulation of plasma insulin concentration after the injection of a large amount of insulin is shown and compared to experimental data [58]. Note that the model does not match the experimental data set. This results from the presence of unmodeled effects at highly supraphysiological insulin concentrations and limitations in the detection quality of the experiment. Therefore, the model is not valid at these extremely high insulin concentrations.

The dynamic insulin degradation behavior of the model was compared to experimentally determined time courses of insulin concentration in plasma after an intravenous insulin injection. Experimental data sets with extremely high [58] and extremely low [59] amounts of injected insulin were used. The extremely low amount (1.65·10^-6 ^*nmol*) was radioactively labeled. Therefore, the dynamics of injected insulin can be tracked though the amount of injected insulin is small compared to endogenous insulin. Measured concentrations of labeled insulin were about 0.5·10^-4 ^*nM *[59] (see Figure 2). The extremely high amount of injected insulin (47.5 *nmol*) resulted in measured insulin concentrations above 1600 *nM *[58] (see Figure 3). Note that the basal concentration of insulin is 0.07 *nM *and peak concentrations in insulin therapy are below 1 *nM *[16,17].

Simulated insulin concentrations for low amounts of injected insulin [59] are relatively close to the experimental data set (Figure 2). When examining Figure 2 on a log-linear scale, it can be seen that the relatively low absolute errors at later points in time correspond to large relative errors (not shown). Note that these high relative errors for low absolute values could at least partly result from manual extraction of the data points from Figure 3 in [59]. As the absolute errors are moderate, Figure 2 is regarded as a qualitative validation of the dynamic model at physiological insulin concentrations.

Simulation results for the injection of unphysiologically high amounts of insulin [58] are not very close to the experimental data set (Figure 3). When examining Figure 3 on a log-linear scale, it can also be seen that the relative errors at later points in time are large (not shown).

Note that an insulin concentration of 1600 *nM *is four orders of magnitude higher than peak concentrations in insulin therapy (below 1 *nM*) and far above physiological values. In this concentration range new unmodeled effects occur. As an example, pinocytosis (fluid-phase endocytosis) significantly contributes to hepatic insulin uptake at high concentrations of insulin [9,60]. In correspondence to nonspecific insulin binding by the liver, nonspecific insulin binding could also occur in other tissues. A result of this additional nonspecific binding would be reversible insulin removal at high insulin concentrations and subsequent insulin release at lower insulin concentrations. Furthermore, the assay of Desbuquois et al. [58] is not able to distinguish between insulin fragments and native insulin. However, after a few minutes, insulin fragments contribute significantly to total insulin, as shown for the injection of small amounts of labeled insulin (see Figure 3 in [59]). Assuming that this holds also for the injection of high amounts of insulin, the assay of Desbuquois et al. overestimates insulin concentrations at later points in time. Note that this is surely not sufficient to explain the whole extent of the error at later points in time.

The effects of pinocytosis and additional nonspecific insulin binding at high insulin concentrations are not quantified in literature and not included in the model. Neglecting these processes (and maybe others that are important at high insulin concentrations) leads to an incorrect model structure for high insulin concentrations. Therefore, the model is not valid at extremely high insulin concentrations.

#### Hepatic insulin receptor internalization

**Figure 4:**
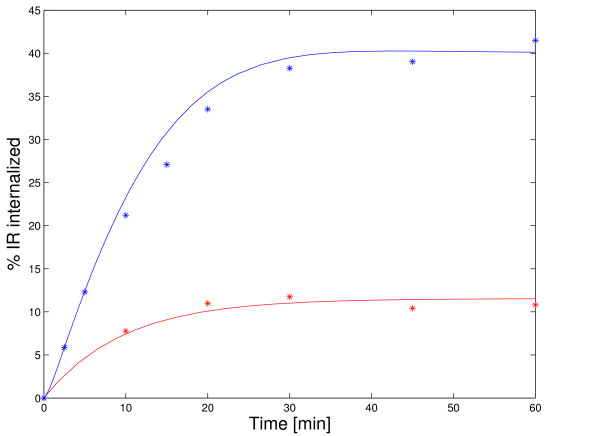
**Dynamic model validation: receptor internalization**. Simulation results for receptor internalization at 100 *nM *insulin (blue) as well as those without insulin (red) are shown and compared to experimental data [34]. Surface receptors were radioactively labeled. This was simulated by setting the initial conditions such that all receptors are in the state *R *at the plasma membrane. Note that the receptor model is linear for constant insulin concentration. Therefore, the assay can be simulated with this choice of initial conditions.

Simulation results for hepatic insulin receptor internalization at 100 *nM *insulin as well as those without insulin were compared to experimental data from literature [34]. As it can be seen in Figure 4, simulation results match the experimental data sets very well. Assuming that simulated insulin binding to the receptor mirrors physiological processes well (we show below that it does), receptor internalization can be used as a direct indicator for hepatic insulin degradation.

It should be stated that the model parameters for receptor internalization and recycling are from the same source as the experimental data sets in Figure 4. Backer et al. took the experimental data sets to estimate the parameters for two separate models of receptor internalization and recycling at 100 *nM *insulin as well as without insulin [34]. We adopted three parameters of these models.

Note that the experimental data sets for receptor internalization result from experiments with Fao cells that are tumor cells of hepatic origin. Though simulations match the experimental data sets almost quantitatively, this can only be regarded as a qualitative model validation for hepatocytes.

### Stationary model validation

**Figure 5:**
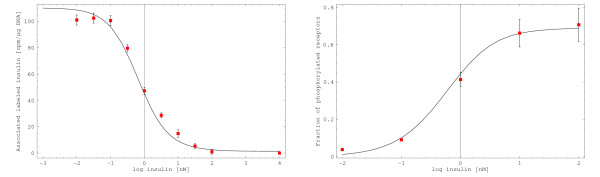
**Stationary model validation: insulin binding and receptor phosphorylation**. **Left**: Cell-associated radioactively labeled insulin is shown as a function of the stationary insulin concentration and compared to experimental data (Figure 4 A in [57]). Almost no labeled insulin should bind to receptors at maximal concentrations of unlabeled insulin. Therefore, the value for the highest concentration of unlabeled insulin was treated as background and subtracted from all values. **Right**: The fraction of phosphorylated receptors is shown as a function of the stationary insulin concentration and compared to experimental data for receptor activation (Figure 4 B in [57]). We regard receptor phosphorylation as a good indicator for receptor activity.

Simulation results for stationary insulin receptor activation and insulin binding were compared to experimental data sets [57]. Klein et al. determined cell-associated radioactively labeled insulin as a function of unlabeled insulin concentration (Figure 4A in [57]). This was done in a competition assay with constant concentration of radioactively labeled insulin and variable concentrations of unlabeled insulin. *In silico *reproduction of this assay and projection of the result on the experimental data set results in a high accordance (Figure 5, left).

Klein et al. also determined the stationary dependency of receptor activity on the insulin concentration (Figure 4B in [57]). We regard receptor activity as an indicator for receptor phosphorylation. Projection of the experimental data set on the stationary fraction of phosphorylated receptors also shows high accordance (Figure 5, right). See Additional file 4 for details about the stationary model validation.

Changes in *t*_*in *_or in the parameters for the pancreas have no effect on the results of stationary model validation, as the system is analyzed at constant insulin concentrations in the stationary case.

Altogether, the model is able to match the experimental data sets for receptor activity and insulin binding very well. Note that the model parameters were not estimated to get these results.

### Model analysis

#### Insulin degradation

The fractions of insulin that are degraded by the liver and the kidney were investigated in several studies. Values for the relative contribution of the liver to insulin degradation in man range from below 50% to 70%, and those for the kidney from 30% to above 50% [5,9,12]. We investigate the reason for this diversity by stationary model analysis.

**Figure 6:**
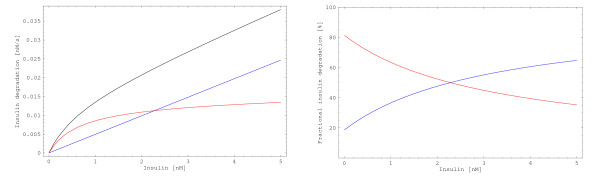
**Renal and hepatic insulin degradation**. **Left**: Stationary insulin degradation rates of the liver (red) and the kidney (blue) and the total insulin degradation rate (black) are shown as functions of insulin concentration. **Right**: Stationary relative contributions of the liver (red) and the kidney (blue) to total insulin degradation depend on the insulin concentration. Note that these fractions are slightly lower in reality. Other tissues, in particular fat and muscle, also contribute to insulin degradation but are not analyzed here. The fractions in this plot refer to the sum of the degradation rates of liver and kidney.

Renal insulin degradation does not saturate [9,47], whereas hepatic insulin degradation saturates [5,9,56]. The physiological situation is mirrored by the model, where hepatic insulin degradation saturates, whereas renal insulin degradation does not (Figure 6). It can be clearly seen that the relative contributions of the liver and the kidney to total insulin degradation strongly depend on the insulin concentration.

In stationary model analysis, the relative contribution of the liver to overall insulin degradation ranges between 81% for insulin concentration tending to zero and 0% for insulin concentration tending to infinity. The relative contribution of the kidney ranges between 19% and 100% (Additional file 4). A significant part of these changes happens beyond physiological insulin levels. However, the fractions vary strongly in the physiological range of insulin concentrations. Between 0 *nM *and 1 *nM *insulin, the relative contribution of the liver is between 81% and 63%, while the contribution of the kidney is between 19% and about 37% (Figure 6 and Additional file 4). Only the liver and the kidney are considered in the analysis of insulin degradation. Other insulin degrading tissues, in particular fat and muscle, are neglected. Therefore, the sum of the relative contributions of the liver and the kidney to insulin degradation is one (100%).

Note that changes in *t*_*in *_or in the parameters for the pancreas do not affect the results of stationary model analysis, as the system is analyzed at constant insulin concentrations. The rate of nonspecific insulin binding equals zero in the unique stationary case. Therefore, it also has no influence on stationary insulin degradation. The stationary analysis of degradation rates and relative contributions to insulin degradation is also independent of the parameter *m*_*body *_(Additional file 4).

Quantitative results regarding relative contributions to insulin degradation are sensitive to changes in the parameter *K kidney*, as increasing *K kidney *by 10% also increases the renal insulin degradation rate (*r*_*kid*_) by 10%. However, changes in *K kidney *only change the values of the relative contributions to insulin degradation but do not lead to a qualitatively different result.

Altogether, relative contributions of the tissues to insulin degradation depend on the insulin concentration. At low insulin concentrations, hepatic insulin degradation is predominant, whereas at high insulin concentrations overall insulin degradation is mainly performed by the kidney. Therefore, different results for the relative contributions of the liver and the kidney to insulin degradation are expected for different experimental settings.

#### Insulin clearance

The quotient of insulin degradation rate and insulin concentration is denoted as insulin clearance *c *[13], which is a widely used quantity to characterize the state of insulin metabolism.

$$c=\frac{(-r_{liv}-r_{kid})\cdot v_{p}}{Ins}$$

The physiological range of insulin clearance in man (70 *kg*) is 700 – 3350 *ml*·*min*^-1 ^[12,13]. We use stationary model analysis to investigate the reason for this diversity.

**Figure 7:**
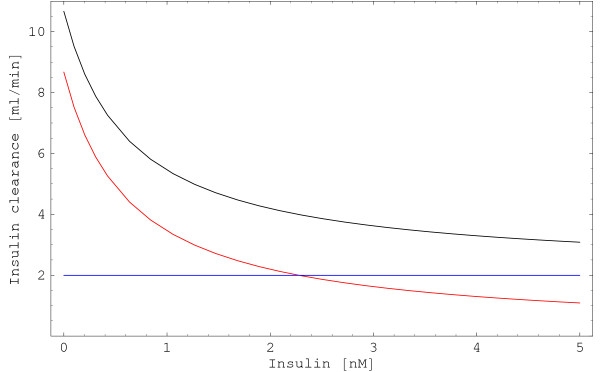
**Renal and hepatic insulin clearance**. Insulin clearance is defined as the quotient of insulin degradation rate and insulin concentration. Total stationary insulin clearance (black) is a function of insulin concentration because hepatic insulin clearance (liver, red) depends on the insulin concentration, whereas renal insulin clearance (kidney, blue) is independent of insulin concentration. A body weight of *m*_*body *_= 200 *g *was used in the computations.

Insulin clearance strongly depends on the insulin concentration (Figure 7). Due to receptor saturation, hepatic insulin clearance decreases for increasing insulin concentrations. The effect of *Ins*^-1 ^in hepatic insulin clearance (-*r*_*liv*_·*v*_*p*_·*Ins*^-1^) strongly dominates the effect of the saturating degradation rate *r*_*liv *_(compare Figures 6, left and 7). Renal insulin clearance is independent of the insulin concentration, as it is a first order process in which the degradation rate is proportional to the insulin concentration. Therefore, the quotient of rate and concentration is constant.

As an example, in a rat whose body weight is 200 *g*, insulin clearance ranges between 10.7 *ml*·*min*^-1 ^for insulin concentration tending to zero and 2.0 *ml*·*min*^-1 ^for insulin concentration tending to infinity (Additional file 4).

Insulin clearance is often used to characterize the state of insulin metabolism. However, its value for analysis of processes that are dominated by saturable components, in particular hepatic insulin degradation, is very limited. A strong dependence on the insulin concentration hampers precise analysis, especially if one cannot guarantee that the insulin concentration is constant during the experiment. This problem does not occur when analyzing first order processes such as renal insulin degradation, where insulin clearance is independent of insulin concentration (Figure 7).

Altogether, the strong dependency of insulin clearance on the insulin concentration is able to explain the wide range of reported values.

#### Parameter estimation

As shown above, insulin degradation at high insulin concentrations is mainly performed by the kidney. Nonspecific insulin binding dampens rapid variations in insulin concentration at all insulin concentrations (Additional file 3). Therefore, the most important parameters at high insulin concentrations are those for the kidney and nonspecific insulin binding.

In order to investigate whether the model structure can reproduce the experimental data set for high amounts of injected insulin [58], we estimated the parameters for the kidney and nonspecific insulin binding. The model with estimated parameter values matches the experimental data set for high amounts of injected insulin significantly better than the model with parameter values from literature (Additional file 3). However, from a physiological point of view, we are convinced that this does not reflect increased model quality. As discussed above, there are unmodeled effects which are not important at physiological insulin concentrations and there is detection of insulin fragments in the assay.

Thus, taking the experimental data set for high amounts of injected insulin [58] to estimate parameter values for our model (Additional file 3) results in parameters that reflect more than the processes they represent. The estimated parameter values also include the effects of processes that are not explicitly described in the model and detection errors of the assay. Therefore, we performed our analysis using the parameter values from literature (Table 1).

#### Sensitivity analysis

We showed that the parameters for the kidney and nonspecific insulin binding are most important at high insulin concentrations (Figure 6 and Additional file 3). Now, we investigate robustness to small changes in these parameters (Additional file 3). Increasing or decreasing the values of *k*1*ub*, *k*2*ub *and *K kidney *by 20% results in moderate differences in simulation results for the injection of high amounts of insulin. Changing *K kidney *by 20% has practically no effect on simulation results for the injection of small amounts of insulin, whereas the effect of small changes (up to 20%) in the parameter values for nonspecific insulin binding is moderate. All simulation results match the experimental data set in an acceptable way (Additional file 3).

On the other hand, the values of *k*1*ub*, *k*2*ub *and *K kidney *cannot be arbitrarily chosen. A nice example for this is that simulation results using an estimated parameter set fail to match the experimental data set for small amounts of injected insulin (Figure 6, top in Additional file 3). This supports our previous argument that the experimental data set for high amounts of injected insulin [58] should not be used for parameter estimation.

The values from literature of *k*1*ub*, *k*2*ub *and *K kidney *are surely not determined with extremely high precision. Small deviations from their nominal values do not lead to dramatic differences in the simulation results for insulin dynamics (Additional file 3), whereas larger changes do. Therefore, the values from literature are at least acceptable estimates for parameterization of the processes they represent.

The parameters for the pancreas (*pansec*, *K pan*, Hill coefficient) are chosen to guarantee that insulin secretion is turned off at peak concentrations in insulin therapy. They have negligible influence on the simulation results for insulin dynamics, as long as the physiological basal level of insulin (0.07 *nM*) is guaranteed by adjusting *pansec *(Additional file 3).

Changes in the parameter *t*_*in *_(for which no value is given) have practically no influence on the simulation results for high amounts of injected insulin [58] (Additional file 3). Simulation results for small amounts of injected insulin [59] are sensitive to changes in *t*_*in*_. If one assumes that *t *= 0 *s *corresponds to the end of insulin injection, simulation results are in each case relatively close to the experimental data set (Additional file 3). However, if one assumes that insulin injection starts at *t *= 0 *s*, simulation results for large injection times are not close to the experimental data set any more (Additional file 3). Unfortunately, the exact procedure of injection is not described in either study [58,59]. We assume a bolus injection at *t *= 0 *s *for both experiments.

Altogether, simulation results for insulin dynamics are sensitive to changes in *t*_*in*_. However, they are robust to changes in the other unknown parameters and in the parameters that are most important at high insulin concentrations.

### Comparison with other models

Comparison of our model predictions for insulin dynamics with those of other models [12-17] (all of which describe insulin dynamics in humans) is only possible if the amount of injected insulin and the model parameters are scaled to reflect differences in body mass between rats and humans. However, compensating the difference between rats and humans solely by scaling body weights and amounts of injected insulin will result in unreliable model predictions because differences in physiology are neglected. Parameter estimation is a possible way of compensating for the differences in physiology between rats and humans, however, experimental data sets used for parameter estimation cannot be reused for the model validation. Therefore, more experimental data sets are necessary to adapt the parameter values from models of insulin dynamics in humans [12-17] to the physiological situation in rats and to perform the model validation with independent data sets.

Estimating the parameters of our model to match experimental data sets for humans as accurately as the models of human insulin dynamics should be possible. The reason for this is that our model considers more processes and therefore has more degrees of freedom. However, the model parameters are not identifiable if only experimental data sets for insulin concentration are used. Therefore, the estimated parameter values are not physiologically relevant.

We investigate whether the analysis of the other models of insulin receptor activation [37,38] leads to the same results as the analysis of our model. The receptor model of Sedaghat et al. [37] shows stationary insulin binding characteristics that match the experimental data set of Klein et al. [57] well, but not as accurately as our model (compare Figure 5 and Additional file 6). Stationary receptor phosphorylation characteristics show significant deviations from the experimental data set (Additional file 6). Note that this only indicates that the model of Sedaghat et al., which describes receptor dynamics in adipocytes, cannot be used for hepatocytes. It should be mentioned that the model of Sedaghat et al. [37] is not suited for stationary model analysis as the total receptor concentration in the stationary case is unphysiologically high. The worst case is a total stationary receptor concentration of 100 *M *in the absence of insulin (Additional file 6). Note that the initial condition for the total receptor concentration (10^-12 ^*M*) is not a stationary solution of the model equations. However, simulation results converge very slowly to the steady state (not shown) as the first order constant for receptor synthesis is about 10^-17 ^*M*·*min*^-1^.

Insulin degradation of adipocytes (i.e. with initial conditions taken from the model of Sedaghat et al.) is five orders of magnitude lower than hepatic insulin degradation (see above). At physiological insulin concentrations, insulin degradation is mainly performed by the liver (Figure 6). Therefore, it is obvious that replacing our receptor model by the model of Sedaghat et al. makes it impossible to match the experimental data set for low amounts of injected insulin.

The situation is different when considering a modified model of Hori et al. [38]. Note that all parameterized models of Hori et al. are only valid at 100 *nM *insulin. Only one of the models of Hori et al. can handle variable insulin concentrations. However, no parameterization is given for this model structure. We parameterized each process of this model structure by taking the average value of the estimated parameter values from the other models of Hori et al. As there are no parameters for insulin binding in the study of Hori et al. [38], we took the parameter values for insulin binding from the model of Wanant et al. [36]. This modified model of Hori et al. is not able to reproduce the stationary insulin binding and receptor activation characteristics from literature as well as our model (compare Figure 4 and Additional file 7). However, all models of Hori et al. reproduce the experimental data set for receptor internalization (compare Figure 5B in [38] and Figure 4). Note that Hori et al. used this data set to estimate model parameters [38].

As the receptor concentrations in the models of Hori et al. [38] are normalized to one, we set the total receptor concentration to 40 *nM *as we do in our model. We replaced the hepatocyte part of our model by the modified model of Hori et al. and left all other processes (nonspecific insulin binding, renal insulin degradation and pancreatic insulin secretion) unchanged. This led to simulation results for low amounts of injected insulin that match the experimental data set [59] about as well as our original model (not shown). The modified model of Hori et al. shows slightly higher hepatic insulin degradation than our model. Therefore, when comparing the simulation results with those of our model, the simulation performs better at the first two points in time, the simulation and our model perform equally well at the third point in time (2 *min*), and our model performs better for the remaining points in time (not shown). For the experiment with high amounts of injected insulin [58], the difference between the models is negligible (not shown) as insulin degradation at high insulin concentrations is mainly performed by the kidney (Figure 6 and Additional file 4).

Note that our model is not able to quantitatively reproduce the dynamic phosphorylation characteristics in endosomes that were measured by Backer et al. in Fao cells [34] and used by Hori et al. [38] for parameter estimation (not shown). However, Fao cells have different physiological characteristics as hepatocytes and therefore quantitative matching of our hepatocyte model to experimental data sets for Fao cells is not necessary. In this case, we rely on the *in vitro *measurements of receptor phosphorylation and dephosphorylation in hepatocytes [51,52] that we used to parameterize our model.

Note that the modified model of Hori et al. corresponds to a reduced model of our receptor model where some parameters and the total receptor concentration are identical.

Both receptor models from literature [37,38] are not able to match all experimental data sets. Therefore, we assume that the additional complexity included in our model, in particular binding of the second insulin molecule, could be necessary. We further investigate this by stationary model analysis. The fraction of insulin receptors with two bound insulin molecules rises with insulin concentration (Additional file 4). At 100 *nM *insulin, which is a common situation in *in vitro *experiments, almost 50% of all receptors have two bound insulin molecules. At the basal insulin concentration, the fraction of receptors with two bound insulin molecules is negligible (0.01%). Therefore, a reduced model structure that neglects all receptor states with two bound insulin molecules is sufficient at low insulin concentrations. However, at insulin concentrations larger that 5 – 10 *nM *insulin receptors with two bound insulin molecules represent a significant fraction and should not be neglected. As in many cases simulation speed is not a limiting factor, we recommend to use our model for small insulin concentrations as well.

### Therapeutic insulin concentrations

The aim of insulin therapy is to achieve sufficient glucose uptake with minimal amounts of insulin [5,10,11]. An interesting question is whether an upper bound for reasonable insulin concentrations exists. We investigate this by combining the results of stationary model analysis and experimental studies from literature.

At about 10 *nM *insulin, the insulin receptor in hepatocytes of rats is almost maximally phosphorylated (Figure 5). Almost 80% of insulin is degraded by the kidney (Additional file 4) and does not contribute to insulin receptor activation. The fraction of insulin that is degraded by the kidney further increases with increasing insulin concentration (Additional file 4).

Half-maximal insulin receptor phosphorylation in rat adipocytes is at 7 ± 1 *nM *insulin (experimental: [61], simulation study: [37]). Glucose uptake in adipocytes is half-maximal at 170 *pM *insulin and saturates at about 3 *nM *insulin [61]. These findings are expected to hold qualitatively also for human adipocytes. As the aim of insulin therapy is to achieve the desired physiological effect (glucose uptake) with minimal amounts of insulin, an upper bound for therapeutic insulin concentrations in man seems to exist. This upper bound is the insulin concentration where a higher insulin concentration does not result in a higher glucose uptake but only leads to increased insulin degradation. Characteristics of glucose uptake in rats imply that this upper bound is at about 3 *nM *and not at about 10 *nM *as implied by receptor phosphorylation characteristics.

Relatively large amounts of insulin or insulin analogues are injected or infused in postprandial glucose control. This mimics the physiological response of healthy individuals [62,63]. Postprandial insulin concentration after a standard meal peaks at 60 – 80 *μU*·*ml*^-1 ^[16,17], which is about 0.35 – 0.5 *nM*. Hepatic insulin degradation is predominant in this concentration interval (Figure 6). Additionally, glucose uptake is strongly, but not fully activated [61]. Overnight control of glucose concentration is performed with basal insulins that show slow absorption kinetics or with continuous injection of short acting insulins [5]. In both cases only insulin concentrations close to the physiological basal concentration are expected.

Therefore, the theoretical upper bound for reasonable therapeutic insulin concentrations in rats (about 3 *nM*) lies significantly above therapeutic insulin levels in humans (about 0.5 *nM*). We suppose that the upper bound for reasonable therapeutic insulin concentrations in man is relatively close to the value postulated for rats.

Altogether, mathematical analysis and experimental results indicate that peak concentrations in insulin therapy are below the upper bound where a higher insulin concentration does not result in a stronger physiological effect.

## Conclusion

We present a detailed dynamic model that describes *in vivo *insulin dynamics and hepatic insulin receptor activation in the rat. Model parameters are taken from *in vitro *experiments and other models. The model is able to reproduce experimental data sets from literature without parameter estimation.

The vast majority of statements about insulin degradation and insulin clearance in the literature is given without explicitly defining the corresponding insulin concentration, and the reported values widely vary. Mathematical analysis shows that relative contributions of the liver and the kidney to total insulin degradation highly depend on the insulin concentration. At low insulin concentrations, insulin is mainly degraded by the liver, whereas renal insulin degradation is predominant at high insulin concentrations. This explains variations in reported values of relative contributions to insulin degradation.

Mathematical analysis also shows that insulin clearance strongly depends on the insulin concentration, which explains variations in reported values. Due to the concentration dependence of insulin clearance, its value for characterizing insulin metabolism is very limited.

The analysis of relative contributions to insulin degradation and the dose-response characteristics of insulin receptor activation and glucose uptake imply the existence of an upper bound for reasonable therapeutic insulin concentrations. Higher insulin concentrations do not result in higher glucose uptake and additional insulin is degraded without having therapeutic effect. However, the upper bound for reasonable therapeutic insulin concentrations is above peak concentrations in insulin therapy.

The detailed model presented here can be used as a starting point for modeling and analysis of the signaling cascades emerging from the hepatic insulin receptor (e.g. MAP kinase cascade and PI3K pathway). This will significantly contribute to understanding the effect of insulin on hepatocytes *in vivo*.

## Methods

### Model parameters and initial conditions

*In vitro *insulin receptor autophosphorylation has a half-life of about 0.5 *min *(Figure 1 in [52]). Assuming first order kinetics, this corresponds to a rate constant of *kyp *= 0.0231 *s*^-1^. *In vitro *insulin receptor dephosphorylation at the plasma membrane has a half-life of about 3 *min *(Figure 2 in [51]). Assuming first order kinetics, this corresponds to a rate constant of *kyd *= 0.00385 *s*^-1^. *In vitro *insulin receptor dephosphorylation at endosomal membranes has a half-life of 1.6 *min *(Figure 2 in [52]). Assuming first order kinetics, this corresponds to a rate constant of *kyden *= 0.00722 *s*^-1^.

We compared weights of livers and bodies given in literature [46]. In average, the liver contributes about 5% to the body weight of rats. There are 10^5 ^insulin receptors per hepatocyte [44]. Assuming that the hepatocyte is a sphere with a diameter of 20 *μm*, this corresponds to a receptor concentration of 40 *nM*. Basal insulin concentration in fasted mice is 0.3 – 0.5 *ng*·*ml*^-1 ^(Gisela Drews, personal communication). As the molecular weight of insulin is 5.7 *kDa*, 0.4 *ng*·*ml*^-1 ^corresponds to 0.07 *nM*. The same basal insulin concentration is assumed for rats. All other parameters were directly taken from the cited references. As discussed above, all parameters are taken from previously published smaller models, *in vitro *experiments or chosen to guarantee the physiological basal level of insulin (0.07 *nM*) and to cut off insulin synthesis at high insulin concentrations. A list of all model parameters is given in Table 1.

Stationary model equations (all derivatives set to zero) were solved for the state variables under the basal insulin concentration as a constraint to get initial conditions that correspond to the basal insulin concentration (Additional files 4 and 5, Table 1). This resulted in a unique solution and was done with the software package *Mathematica *(Wolfram Research).

### Dynamic model validation

Kruse et al. [59] used rats with a body weight of 238 ± 20 *g*. Insulin injection was 100 *μl *of 12 – 21 *pM *radioactively labeled insulin. A body weight of *m*_*body *_= 238 *g *and an insulin injection of n_*,*in *_= 100 *μl*·16.5 *pM *= 1.65·10^-6 ^*nmol *and *n*_*in *_= 0 *nmol *was used for the simulation. The experimental data set is given in % dose per *ml *serum. Multiplied by the amount of injected labeled insulin and divided by 100, these values give the concentrations of labeled insulin in plasma [*nmol/ml*]. Desbuquois et al. [58] used rats with a body weight of 180 – 200 *g*. Insulin injection was 25 *nmol*/100 *g *body weight. A body weight of *m*_*body *_= 190 *g *and an insulin injection of *n*_*in *_= 47.5 *nmol *and n_*,*in *_= 0 *nmol *was used for the simulation. The experimental data set is already given as concentration values. As the exact procedure of injection is not described in either study, we assumed that the injection was given as a bolus at *t *= 0 *s*.

Backer et al. investigated receptor internalization in Fao hepatoma cells [34]. Surface receptors were radioactively labeled at low temperature (on ice), stopping receptor internalization. Incubation at 37°C initiated receptor internalization in the assay. This experiment was simulated by setting initial conditions such that all receptors are at the surface (*R *= 40). If the insulin concentration is constant, the receptor model is linear and the superposition principle holds. Therefore, the assay can be simulated with this choice of initial conditions.

Dynamic simulation was performed with the software package MATLAB (The MathWorks). The executable *in vivo *and *in vitro *models are given in MATLAB format in Additional files 1 and 2, respectively.

### Stationary analysis

The liver is a dynamic system. Its physiological state and its contribution to insulin degradation depend on the insulin concentration. However, the rate of hepatic insulin degradation is not uniquely defined by the present insulin concentration. The hepatic insulin degradation rate strongly depends on the amount of free receptors at the plasma membrane. Assume that the system is not in steady state. In this case, for the model, insulin concentrations from all points in time since the system left the steady state affect the physiological state of the liver and in particular the amount of free insulin receptors at the plasma membrane.

The steady state of our model is uniquely defined by the steady state insulin concentration. Note that the stationary case corresponds to the steady state of the system. In the stationary case, all characteristics of the system (e.g. insulin degradation rates, insulin clearance, receptor activation or insulin binding) can be expressed as functions of the stationary insulin concentration. Thus, stationary analysis gives insights into the system that are not biased by dynamic effects.

All stationary computations were performed with the software package *Mathematica *(Wolfram Research) and are given in Additional files 4, 6, and 7. Additional file 5 is a PDF version of Additional file 4.

## Abbreviations

ass: assumption; bpm, beats per minute; calc: calculated; ml: milliliter (10^-3 ^*l*); nM: nano molar (10^-9 ^*mol*·*l*^-1^); nmol: nano mol (10^-9 ^*mol*); ODE: ordinary differential equation; EN: in endosomes; PM: at the plasma membrane; I1: one insulin molecule bound to the receptor; I2: two insulin molecules bound to the receptor.

## Authors' contributions

MK developed the model, performed the analysis and drafted the manuscript. EDG initiated and supervised the study.

## Supplementary Material

Additional file 1 *In vivo *model. This file contains a MATLAB model to simulate *in vivo *experiments. Click here for file

Additional file 2 *In vitro *receptor model. This file contains the receptor part of the *in vivo *model and can be taken to simulate *in vitro *experiments with a constant insulin concentration. Click here for file

Additional file 3 Robustness and parameter estimation. This file describes the examination of robustness to changes in parameter values and the estimation of the model parameters. Click here for file

Additional file 4 Stationary model analysis. In this file, the stationary model equations are analyzed. Click here for file

Additional file 5 Stationary model analysis in portable document format. This file is equivalent to Additional file 4 but results from a conversion to PDF. Click here for file

Additional file 6 Stationary analysis of the model of Sedaghat et al. The stationary model equations of Sedaghat et al. [37] are analyzed. Click here for file

Additional file 7 Stationary analysis of a modified model of Hori et al. The stationary model equations of a modified model of Hori et al. [38] are analyzed. Click here for file
