# Supplementary material for: Mathematical modeling and analysis of insulin clearance in vivo
Source: BMC Syst Biol. 2008 May 13;2:43. doi: 10.1186/1752-0509-2-43 (PMC2430945; doi:10.1186/1752-0509-2-43)
Supplement: Additional file 3 — Robustness and parameter estimation. This file describes the examination of robustness to changes in parameter values and the estimation of the model parameters. [file 1752-0509-2-43-S3.pdf]

Additional file 3:
Examination of robustness and parameter estimation

Simulation results for insulin dynamics were compared to experimental data sets for low [1] and high [2] amounts of injected insulin. The presence of unmodeled effects at highly unphysiological insulin concentrations (which corresponds to an incorrect model structure for these insulin concentrations) and the detection of insulin fragments by the assay explain why simulation results are not close to the experimental data set for high amounts of injected insulin.
In the first part of this Additional file, the effects of small changes in the parameters for which no value was found in literature and in parameters that are important at high insulin concentrations are investigated. In the second part, values are estimated for parameters that are important at high insulin concentrations such that simulation results for high amounts of injected insulin [2] match the experimental data set as accurately as possible.

Examination of robustness

Injection time

The duration of insulin injection t_{in} was not given in the description of the experimental data sets for insulin dynamics [1, 2]. Therefore, a bolus injection ($t_{in} = 0\ s$) was assumed at $t = 0\ s$. Simulation results for different values of t_{in} ($0\ s \leq t_{in} \leq 45\ s$) show significant differences for low amounts of injected insulin and relatively small differences for high amounts of injected insulin (Figure 1). If one assumes that $t = 0\ s$ corresponds to the end of insulin injection, the simulation matches the experimental data set for low amounts of injected insulin [1] in an acceptable way for each chosen value of t_{in}. However, if one assumes that $t = 0\ s$ corresponds to the beginning of insulin injection, the simulation results do not match the experimental data for increasing values of t_{in}. Note that the amount of injected insulin is only given as an interval [1]. The simulations in all figures were performed with the mean value of injected insulin amounts.
Altogether, simulation results are sensitive to changes in the injection time t_{in}.

Parameters for the pancreas

Pancreatic insulin secretion is realized by the rate r_{pan}.

$$r_{pan} = pansec \cdot \left(1 - \frac{Ins^{ch}}{Ins^{ch} + Kpan^{ch}}\right)$$

The parameter $Kpan$ represents the insulin concentration of half-maximal insulin secretion and was chosen as $Kpan = 0.5\ nM$. The Hill coefficient ch determines how sharply insulin secretion is cut off at the insulin concentration $Kpan$. As the basal concentration of insulin is $0.07\ nM$, values of $Kpan$ smaller than $0.1\ nM$ are surely not physiological and as such were not investigated. Peak concentrations in insulin therapy are below $1\ nM$ [3, 4]. Therefore, values of $Kpan$ greater than $10^4\ nM$ are surely unrealistic. Figure 2 shows the simulation results for both experimental data sets for different values of $Kpan$ ($0.1\ nM \leq Kpan \leq 10^4\ nM$). It can be seen that changing $Kpan$ over five orders of magnitude has no influence on the simulation results for insulin dynamics. Changing the Hill coefficient ch in the interval $1 \leq ch \leq 50$ also has almost no effect on the simulation results for insulin dynamics (Figure 3). Michaelis-Menten kinetics of insulin secretion correspond to

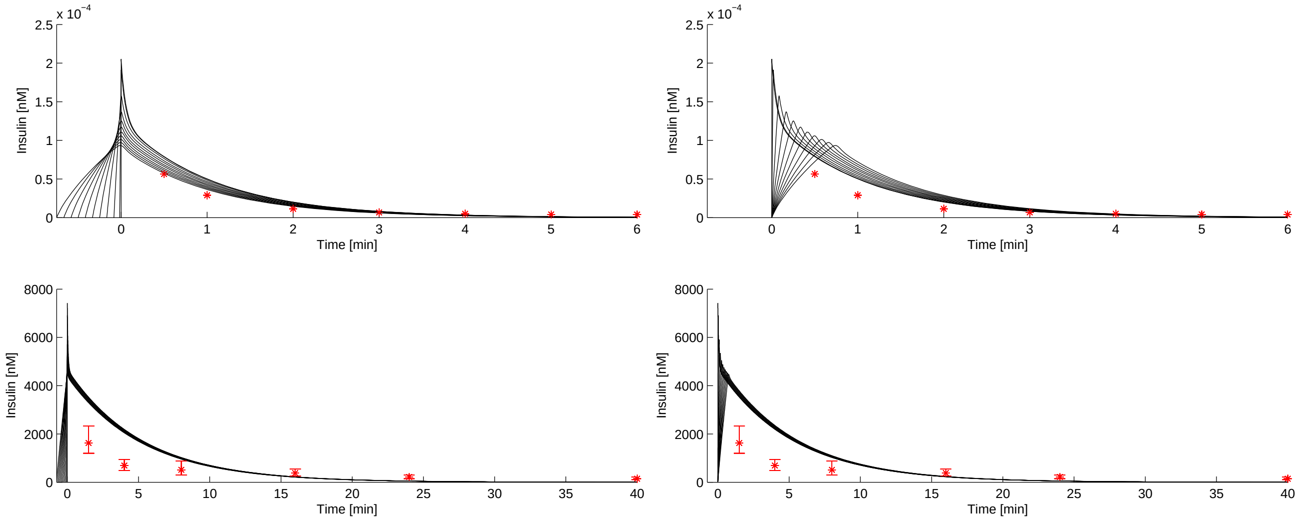

Figure 1: Sensitivity to changes in t_{in}

The value of t_{in} was varied in the interval $0\ s \leq t_{in} \leq 45\ s$. In the plots on the left hand side, the point in time $t = 0\ s$ denotes the end of insulin injection. Therefore, simulations start with the beginning of insulin injection at $t = -t_{in}$. In the plots on the right hand side, the point in time $t = 0\ s$ denotes the beginning of insulin injection.

Top: Injection of low amounts of radioactively labeled insulin [1].

Bottom: Injection of high amounts of insulin [2].

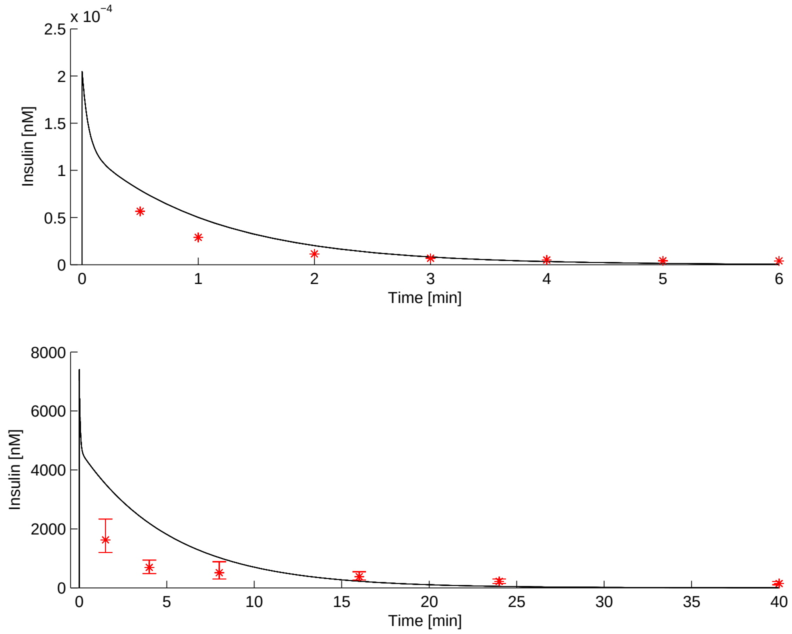

Figure 2: Sensitivity to changes in $Kpan$

The value of $Kpan$ was varied in the interval $0.1\ nM \leq Kpan \leq 10^4\ nM$. Ten simulations covering the entire interval $(0.1\ nM, 0.2\ nM, 0.5\ nM, 0.8\ nM, 1\ nM, 10\ nM, 100\ nM, 1000\ nM, 1000\ nM, 10000\ nM)$ are shown. The differences vanish due to thickness of the lines.

Top: Injection of low amounts of radioactively labeled insulin [1].

Bottom: Injection of high amounts of insulin [2].

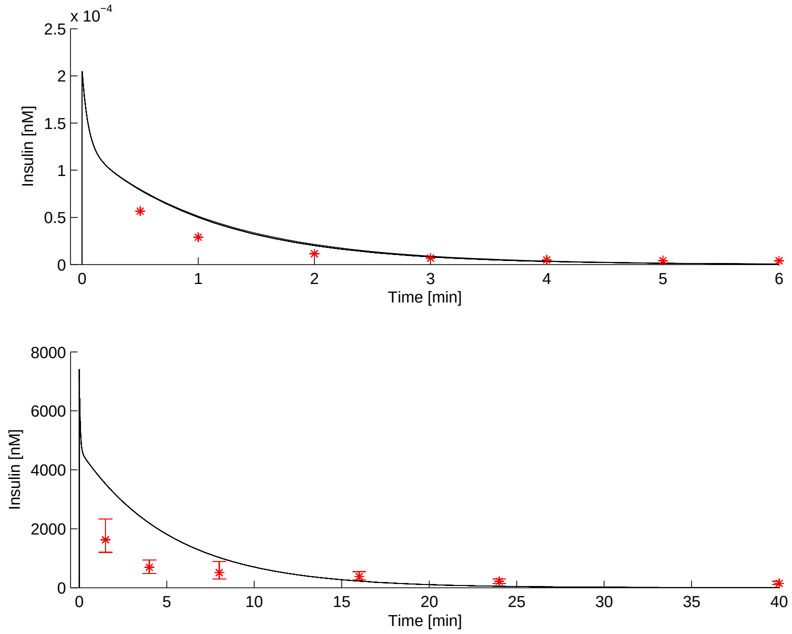

Figure 3: Sensitivity to changes in the Hill coefficient ch
The value of the Hill coefficient ch was varied in the interval $1 \leq ch \leq 50$. Ten simulations covering the entire interval $(1, 5, 8, 10, 12, 15, 20, 30, 40, 50)$ are shown. The differences vanish due to thickness of the lines.
Top: Injection of low amounts of radioactively labeled insulin [1].
Bottom: Injection of high amounts of insulin [2].

$ch = 1$, whereas $ch = 50$ corresponds to a very sharp cut-off of insulin secretion at the insulin concentration $Kpan$.
The low sensitivity of simulation results to changes in the parameters for the pancreas can be easily explained. When injecting small amounts of insulin [1], insulin concentration is only slightly increased in comparison to the basal level. In this concentration range insulin secretion is still turned on for realistic parameter values. When injecting very high amounts of insulin [2], the amount of secreted insulin is small compared to injected insulin. The maximal insulin dose secreted by the pancreas in ten minutes corresponds to 1 nM insulin (Additional file 4), whereas the injected insulin dose corresponds to $n_{in}/v_p \approx 7400\ nM$. It can easily be seen that the influence of pancreatic insulin secretion is also negligible in this case.
Altogether, Figures 2 and 3 show that simulation results for insulin dynamics are robust to changes in the unknown parameters for the pancreas.

Parameters for the kidney and nonspecific insulin binding

At low insulin concentrations, insulin degradation is mainly performed by the liver, whereas renal insulin degradation is predominant at high insulin concentrations (see manuscript). As we show below, nonspecific insulin binding dampens changes in insulin concentration at all insulin concentrations. Thus, deviations of the simulation results from the experimental data set for high amounts of injected insulin mainly result from the parameters for nonspecific insulin binding ($k1ub$ and $k2ub$) and the kidney ($Kkidney$).
The parameters $Kkidney$, $k1ub$ and $k2ub$ were varied over an interval of 80 % to 120 % of the parameter values from literature to investigate whether small errors in these parameter values are able to explain the differences between simulation results and experimental data sets for insulin dynamics.
Simulation results for high amounts of injected insulin show moderate sensitivity to small changes in the parameter $Kkidney$, whereas simulation results for low amounts of injected insulin are not sensitive to small changes in $Kkidney$ (Figure 4). Simulation results for both experimental data sets show moderate sensitivity to small changes in the parameters for nonspecific insulin binding (Figure 5). This shows that nonspecific insulin binding dampens rapid changes in insulin concentration at all insulin concentrations.

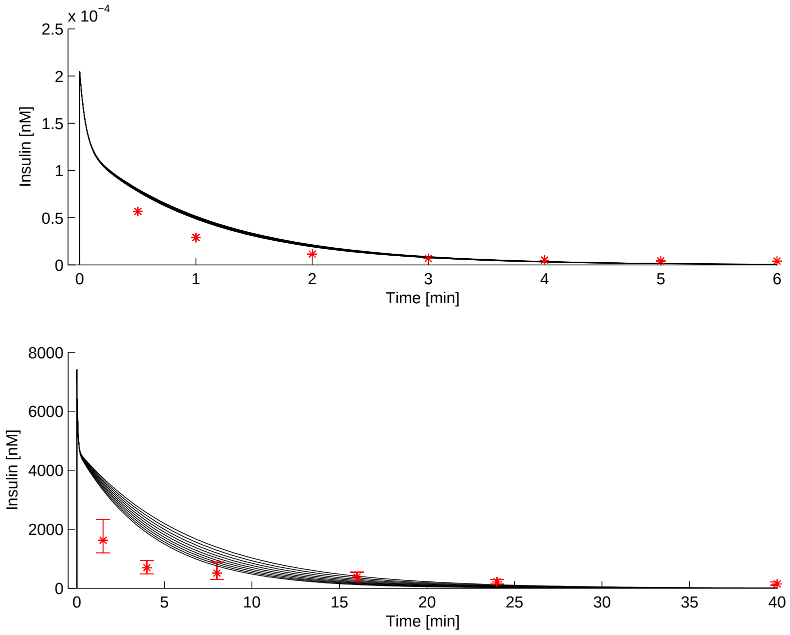

Figure 4: Sensitivity to changes in $Kkidney$
The value of $Kkidney$ was varied in the interval $0.8 \cdot Kkidney \leq Kkidney \leq 1.2 \cdot Kkidney$.
Top: Injection of low amounts of radioactively labeled insulin [1].
Bottom: Injection of high amounts of insulin [2].

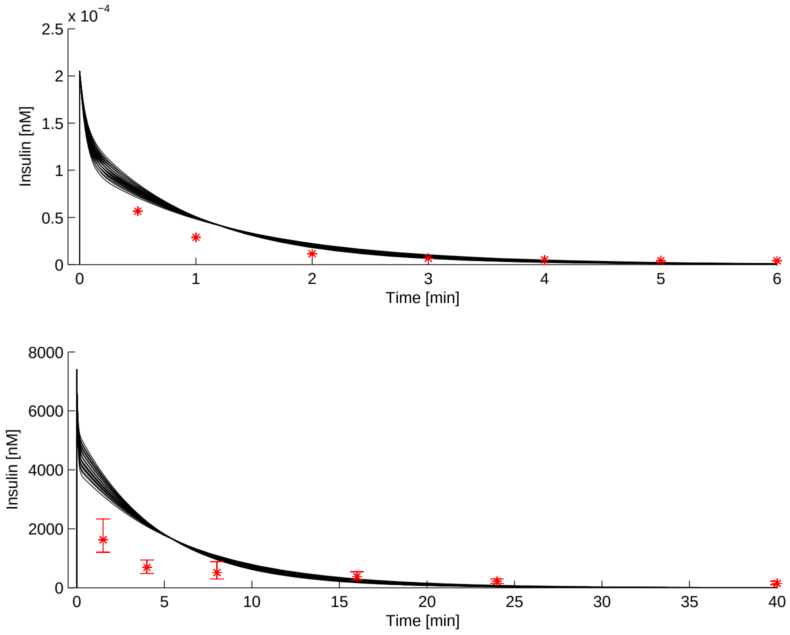

Figure 5: Sensitivity to changes in the parameters for nonspecific insulin binding
The values of $k1ub$ and $k2ub$ were simultaneously varied in the intervals $0.8 \cdot k1ub \leq k1ub \leq 1.2 \cdot k1ub$ and $0.8 \cdot k2ub \leq k2ub \leq 1.2 \cdot k2ub$. Simulations for each combination of $(0.8, 0.9, 1, 1.1, 1.2) \cdot k1ub$ and $(0.8, 0.9, 1, 1.1, 1.2) \cdot k2ub$ are shown.
Top: Injection of low amounts of radioactively labeled insulin [1].
Bottom: Injection of high amounts of insulin [2].

Parameter estimation

The model with parameter values from literature shows significant deviations from the experimental data set for high amounts of injected insulin. Renal insulin degradation is predominant at the resulting high insulin concentrations. Insulin concentration in the assay with low amounts of injected insulin [1] is low and within the range where hepatic insulin degradation is predominant. In addition, the simulation with parameter values from literature is relatively close to the experimental data set and the liver subsystem is also validated by several other experimental data sets (see manuscript). As shown above, nonspecific insulin binding dampens changes in insulin concentration at all insulin concentrations. Thus, the parameters for nonspecific insulin binding ($k1ub$ and $k2ub$) and the kidney ($Kkidney$) are important at high insulin concentrations. Their values were estimated using the experimental data set for high amounts of injected insulin [2]. Note that this data set cannot be used for the model validation if it was already used for parameter estimation.
Taking all dynamic experimental data sets for parameter estimation is theoretically also possible. This was not done here, as in this case there would be no experimental data left to perform the dynamic model validation.
Optimization was performed using the *lsqnonlin* routine from MATLAB. The parameter values were varied in the intervals $0.01\ s^{-1} \leq k1ub \leq 10\ s^{-1}$, $0.01\ s^{-1} \leq k2ub \leq 10\ s^{-1}$ and $0.0225 \cdot 10^{-3}\ l \cdot (s \cdot g)^{-1} \cdot m_{kidney} \leq Kkidney \leq 10^{-3}\ l \cdot (s \cdot g)^{-1} \cdot m_{kidney}$.
The parameters $k1ub$, $k2ub$ and $Kkidney$ were estimated to minimize two different score functions separately.

$$ssq = \sum_{i=1}^{n} (Ins_{sim,i} - Ins_{data,i})^2$$

$$nsq = \sum_{i=1}^{n} \left(\frac{Ins_{sim,i} - Ins_{data,i}}{Ins_{data,i}} \right)^2$$

Minimizing the sum of squares ssq of the differences between simulated insulin concentration $Ins_{sim,i}$ and measured insulin concentration $Ins_{data,i}$ resulted in a different parameter set than that from minimizing the normalized sum of squares nsq where the differences between simulated and measured insulin concentrations are divided by the corresponding measured insulin concentrations (Table 1).
Simulation results using the estimated parameters are closer to the experimental data set for high amounts of injected insulin than the simulation results using values from literature for these parameters (Figure 6, bottom). The experimental data set for low amounts of injected labeled insulin [1] was taken as the dynamic model validation (Figure 6, top). It can be seen that simulation results using values from literature for $k1ub$, $k2ub$ and $Kkidney$ are closer to the experimental data set than simulation results using parameters that minimize the nsq score function. Thus, the estimated parameters that result from using the nsq score function can be rejected. Simulation results using parameter values from literature are comparably close to the experimental data set as simulation results using parameters that minimize the ssq score function. Therefore, the estimated parameters that result from using the ssq score function cannot be rejected.
The parameter $Kkidney$ was also estimated without changing the remaining parameters (Table 1). The allowed interval was $0.0225 \cdot 10^{-3}\ l \cdot (s \cdot g)^{-1} \cdot m_{kidney} \leq Kkidney \leq 10^{-3}\ l \cdot (s \cdot g)^{-1} \cdot m_{kidney}$. Simulation results using estimated values for $Kkidney$ are closer to the experimental data set for high amounts of injected insulin than simulation results using the value from literature (Figure 7, bottom). The experimental data set for low amounts of injected labeled insulin [1] was again taken as the dynamic model validation (Figure 7, top). Simulation results using both estimated parameter sets are slightly closer to the experimental data set than simulation with the value from literature for $Kkidney$. However, the difference is small as the kidney has little influence on insulin degradation at low insulin concentrations.
All estimated values for $Kkidney$ are greater than the value from literature (Table 1), the maximal value is about three times greater than the value from literature. This reflects the inclusion of unmodeled additional insulin degradation (e.g. by pinocytosis) into the degradation rate of the kidney. The estimated values for $k1ub$ are about three times greater than the value from literature. This reflects the inclusion of unmodeled additional nonspecific binding by other tissues into the hepatic rate of nonspecific insulin binding. The value of $k2ub$ is decreased by a factor of 1.6 or a factor of 3.5, which leads to delayed release of nonspecifically bound insulin. This results in higher insulin concentrations at later points in time, which can be clearly seen in Figure 6 (bottom, nsq). However, as discussed in the manuscript, these increased insulin concentrations at later points in time result at least partly from the detection of insulin fragments.
Thus, limitations in the detection quality of the assay are one reason for relatively low values of $k2ub$ in parameter estimation. As simulations using the ssq parameter values and values from literature (Table 1) are comparably

Table 1: Results of parameter estimation
The initial condition of nonspecifically bound insulin is $Ins_{ub} = 1.29948 \cdot 10^{-6} \cdot m_{body}$ for values from literature of $k1ub$ and $k2ub$. The initial condition of nonspecifically bound insulin is $Ins_{ub} = 0.07 * k1ub * v_d/k2ub$ for arbitrary values of these parameters (see Additional file 4). The value of $Kkidney$ in each case is obtained by multiplying the value in the table by $factor$ ($factor = 10^{-3}\ l \cdot (s \cdot g)^{-1} \cdot m_{kidney}$). ssq and nsq refer to the score function that is minimized during the optimization. Only the value of $Kkidney$ was varied in the optimizations that result in the parameter sets ssq-$Kkidney$ and nsq-$Kkidney$.

Parameter	ssq	nsq	ssq-$Kkidney$	nsq-$Kkidney$	Value from literature	Source
$Kkidney \cdot \frac{1}{factor}$	0.0485	0.0318	0.0726	0.0521	0.0225	[5]
$k1ub$	1.0541 s^{-1}	1.3109 s^{-1}	0.35 s^{-1}	0.35 s^{-1}	0.35 s^{-1}	[6]
$k2ub$	0.1216 s^{-1}	0.0569 s^{-1}	0.2 s^{-1}	0.2 s^{-1}	0.2 s^{-1}	[6]

close to the experimental data set for low amounts of injected insulin (Figure 6, top), values of $k1ub$ greater than the value from literature and values of $k2ub$ smaller than the value from literature cannot be rejected.
Figures 6 and 7 clearly show that nonspecific insulin binding dampens rapid changes in insulin concentration (see also Figure 5). With increased nonspecific insulin binding and decreased insulin release (Table 1), the nsq parameter set for estimating $k1ub$, $k2ub$ and $Kkidney$ results in decreased insulin concentrations at early points in time and prolonged presence of increased insulin concentrations (Figure 6). The strong effect on simulation results for low amounts of injected insulin is mainly performed by nonspecific insulin binding as simulation results for low amounts of injected insulin are not sensitive to changes in $Kkidney$ (Figures 4 and 7).
Altogether, it was possible to get simulation results much closer to the experimental data set for high amounts of injected insulin by changing $k1ub$, $k2ub$ and $Kkidney$ (Figure 6) or only $Kkidney$ (Figure 7). The nsq parameter set could be rejected as simulation results using this parameter set fail to match the experimental data set for low amounts of injected insulin. For reasons discussed in the manuscript, we recommend that values from literature should be used for all parameters when using the model for physiological insulin concentrations.

References

[1] Kruse V, Jensen I, Permin L, Heding A: **Fate of insulin analogs in intact and nephrectomized rats determined by their receptor binding constants.** *Am J Physiol* 1997, **272**(6 Pt 1):E1089–98.

[2] Desbuquois B, Lopez S, Burlet H: **Ligand-induced translocation of insulin receptors in intact rat liver.** *J Biol Chem* 1982, **257**(18):10852–60.

[3] Wilinska ME, Chassin LJ, Schaller HC, Schaupp L, Pieber TR, Hovorka R: **Insulin kinetics in type-I diabetes: continuous and bolus delivery of rapid acting insulin.** *IEEE Trans Biomed Eng* 2005, **52**:3–12.

[4] Puckett W, Lightfoot E: **A model for multiple subcutaneous insulin injections developed from individual diabetic patient data.** *Am J Physiol* 1995, **269**(6 Pt 1):E1115–24.

[5] Schlatter E, Schurek H, Zick R: **Renal handling of homologous and heterologous insulin in the isolated perfused rat kidney.** *Pflugers Arch* 1982, **393**(3):227–31.

[6] Hammond B, Christensen J, Smith G: **A model of insulin distribution and uptake in the perfused rat liver.** *J Theor Biol* 1991, **149**:121–39.

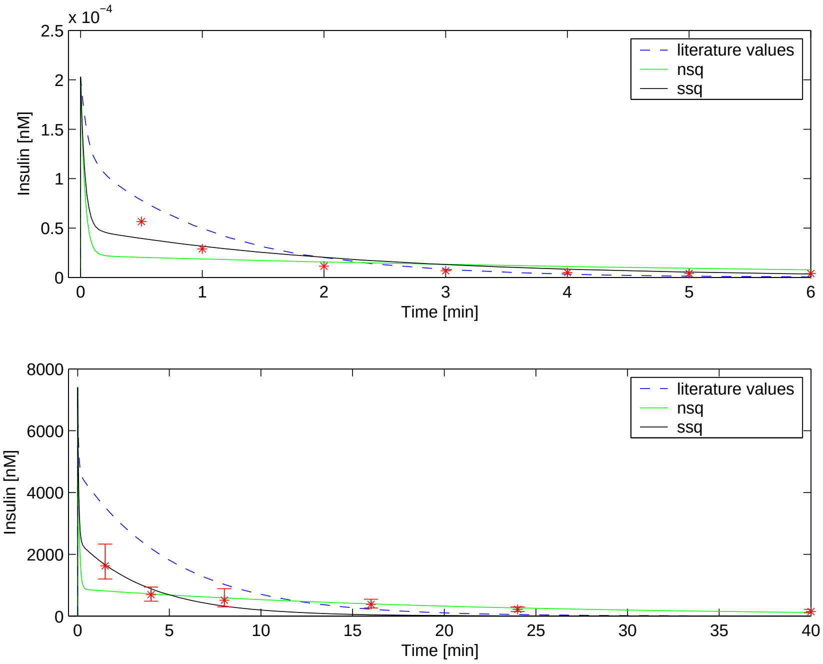

Figure 6: Simulation results with estimated values for $k1ub$, $k2ub$ and $Kkidney$
See Table 1 for estimated parameter values. nsq and ssq refer to the minimized score function.
Top: Injection of low amounts of radioactively labeled insulin [1].
Bottom: Experimental data set [2] used for parameter estimation and simulation results.

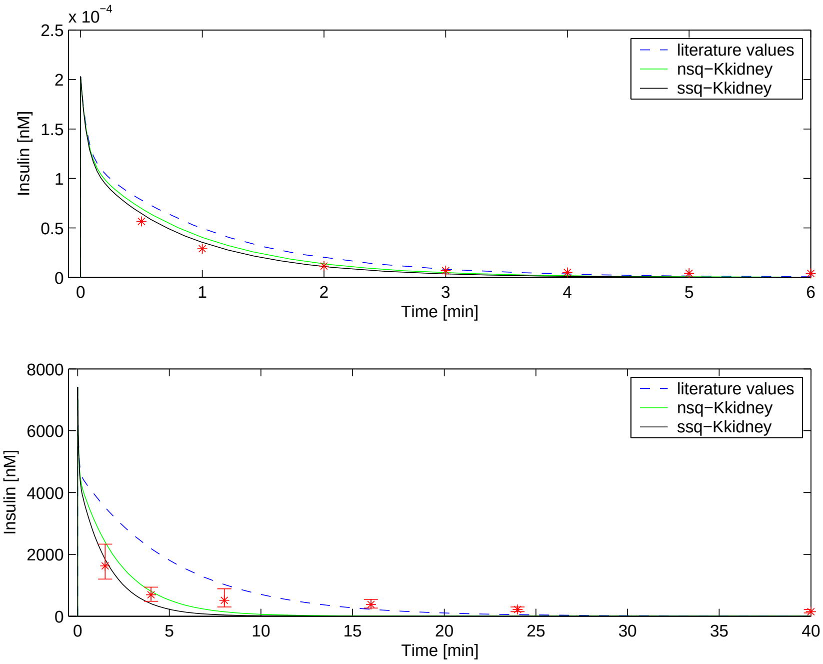

Figure 7: Simulation results with estimated values for $Kkidney$
See Table 1 for estimated parameter values. nsq and ssq refer to the minimized score function.
Top: Injection of low amounts of radioactively labeled insulin [1].
Bottom: Experimental data set [2] used for parameter estimation and simulation results.
