# Supplementary material for: Mathematical modeling and analysis of insulin clearance in vivo
Source: BMC Syst Biol. 2008 May 13;2:43. doi: 10.1186/1752-0509-2-43 (PMC2430945; doi:10.1186/1752-0509-2-43)
Supplement: Additional file 5 — Stationary model analysis in portable document format. This file is equivalent to Additional file 4 but results from a conversion to PDF. [file 1752-0509-2-43-S5.pdf]

```
(* Stationary analysis of insulin clearance *)

(* All rates are in nmol/(l*s); all concentrations in nM;
 all amounts of substances in nmol; all volumes in l *)

Param = {kins → 10^(-3), kins1d → 4 * 10^(-4), kins2d → 4 * 10^(-2),
   kins1den → 1.925 * 10^(-3), kins2den → 3.85 * 10^(-3), kyd → 0.00385, kyden → 0.00722,
   kyp → 0.0231, intk1 → 5.5 * 10^(-4), intk2 → 2 * 10^(-4), reck1 → 1.533 * 10^(-3)};
Rtotal = 40;
ReceptorBalance =
  {I2Rpen → Rtotal - R - IR - I2R - Rp - IRp - I2Rp - Ren - IRen - I2Ren - Rpen - IRpen};

Rates = {r1 → kins * R * Ins - kins1d * IR, r2 → kins * Rp * Ins - kins1d * IRp,
   r3 → kins * IR * Ins - kins2d * I2R, r4 → kins * IRp * Ins - kins2d * I2Rp,
   r5 → kyd * Rp, r6 → kyp * IR - kyd * IRp, r7 → kyp * I2R - kyd * I2Rp,
   i1 → kins1den * IRen, i2 → kins1den * IRpen, i3 → kins2den * I2Ren,
   i4 → kins2den * I2Rpen, i5 → kyden * Rpen, i6 → kyp * IRen - kyden * IRpen,
   i7 → kyp * I2Ren - kyden * I2Rpen, f1 → intk2 * R - reck1 * Ren, f2 → intk2 * IR,
   f3 → intk2 * I2R, f4 → intk1 * Rp - reck1 * Rpen, f5 → intk1 * IRp, f6 → intk1 * I2Rp};

Liver = {-r1 + r5 - f1 == 0, r1 - r3 - r6 - f2 == 0, r3 - r7 - f3 == 0,
   -r2 - r5 - f4 == 0, r2 - r4 + r6 - f5 == 0, r4 + r7 - f6 == 0, i1 + i5 + f1 == 0,
   -i1 + i3 - i6 + f2 == 0, -i3 - i7 + f3 == 0, i2 - i5 + f4 == 0, -i2 + i4 + i6 + f5 == 0 };

RatesParametersBalance = Rates /. ReceptorBalance /. Param;

LiverODE = Liver /. RatesParametersBalance;

Circulation = {k1ub -> 0.35, k2ub -> 0.2,
   RhoLiver -> 1.051 * 10^3,
   Mliver -> 0.05 * bw,
   Vhep -> (Mliver / RhoLiver) * 0.78,
   Vdisse -> 0.272 * Vhep * RhoLiver * 10^(-3),
   Vplasma → 0.03375 * bw * 10^(-3),
   Mkidney -> 2 * 0.85 * bw / 230,
Kkidney -> (0.0225 / 10^3) * Mkidney} ;


RateLiver = -(-r1 - r2 - r3 - r4) * Vhep / Vplasma //. Circulation; (*nmol/(l*s)*)
RateKidney = Kkidney * Ins / Vplasma //. Circulation;  (*nmol/(l*s)*)

ErgLiver = Solve[LiverODE, {R, IR, I2R, Rp, IRp, I2Rp, Ren, IRen, I2Ren, Rpen, IRpen}];

RateLiverFinished = RateLiver /. RatesParametersBalance /. ErgLiver(*nmol/(l*s)*);

RateTotal = RateLiverFinished + RateKidney;


(* Initial conditions for simulation *)

ErgLiverBasal = ErgLiver /. Ins → 0.07
I2RpenBasal → Rtotal - R - IR - I2R - Rp - IRp - I2Rp - Ren - IRen - I2Ren - Rpen - IRpen /.
 ErgLiverBasal

pansec =
 (RateLiverFinished + RateKidney) / (1 - Ins^n / (Ins^n + ks^n)) /. Ins → 0.07 /. ks → 0.5 /.
  n → 10


{{I2Ren → 0.000121295, IRen → 0.145537, Ren → 4.88528,
  IRpen → 0.492464, Rpen → 0.122602, R → 31.619, Rp → 0.227528,
  IRp → 2.07275, IR → 0.430007, I2R → 0.000696311, I2Rp → 0.00363012}}

{I2RpenBasal → 0.000433466}

{0.0016976}
```

```
(* Initial condition for Ins_ub as a function of k1ub and k2ub *)

equbstat = {0 == (k1ub * Ins * Vdisse - k2ub * Insub) / (Vplasma)};
InsUbstat = Flatten[Solve[equbstat, Insub]];

(*general case*)
InsUbstat /. Ins → 0.07
(*for literature values of k1ub and k2ub*)
InsUbstat /. Ins → 0.07 //. Circulation
```

$\left\{\text{Insub} \to \frac{0.07\ \text{k1ub Vdisse}}{\text{k2ub}}\right\}$

$\{\text{Insub} \to 1.29948 \times 10^{-6}\ \text{bw}\}$

```
(* Insulin secretion at basal insulin concentration [nM/s] *)
InsSecBas = RateLiverFinished + RateKidney /. Ins → 0.07
(* Maximal insulin secretion in 10 minutes [nM] *)
InsSecBas * 60 * 10
```

{0.0016976}

{1.01856}

```
(* Insulin removal rates *)

FigRates = Plot[{RateTotal, RateLiverFinished, RateKidney}, {Ins, 0, 5},
  PlotStyle → {RGBColor[0, 0, 0], RGBColor[1, 0, 0], RGBColor[0, 0, 1]},
  FrameLabel → {"Insulin [nM]", "Insulin degradation [nM/s]"},
  (*RotateLabel→False,*) Frame → True, PlotRange → {All, All}, ImageSize → 400]
```

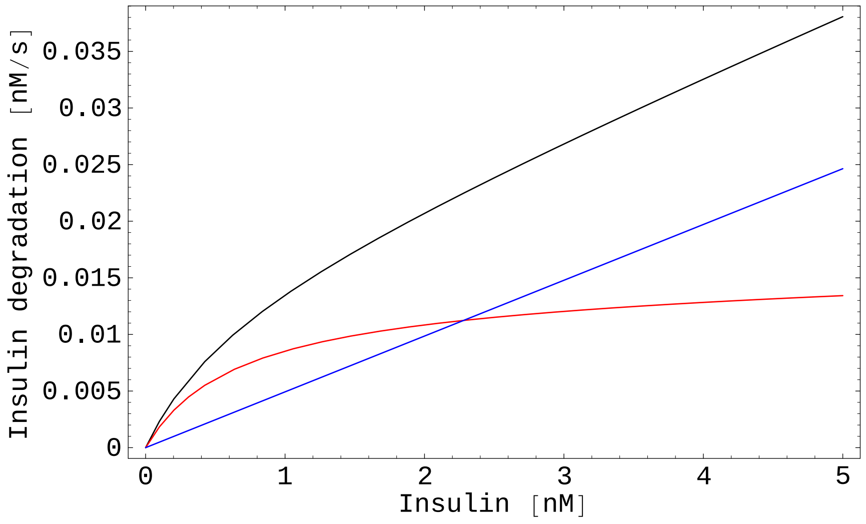

- Graphics -

```
(* Export["ins_rates_stat.eps",FigRates,"EPS",
  ConversionOptions→ {"IncludeSpecialFonts"→ True}] *)
```

```
(* Fractional contribution to insulin removal *)

FracLiver = RateLiverFinished / (RateLiverFinished + RateKidney);
FracKidney = RateKidney / (RateLiverFinished + RateKidney);

FigFrac = Plot[{100 * FracLiver, 100 * FracKidney},
  {Ins, 0, 5}, PlotStyle → {RGBColor[1, 0, 0], RGBColor[0, 0, 1]},
  FrameLabel → {"Insulin [nM]", "Fractional insulin degradation [%]"},
  Frame → True, PlotRange → {All, {0, 100}}, ImageSize → 400]

Limit[FracLiver, Ins → 0]
Limit[FracLiver, Ins → Infinity]

FracKidney /. Ins → 10
FracKidney /. Ins → 1
```

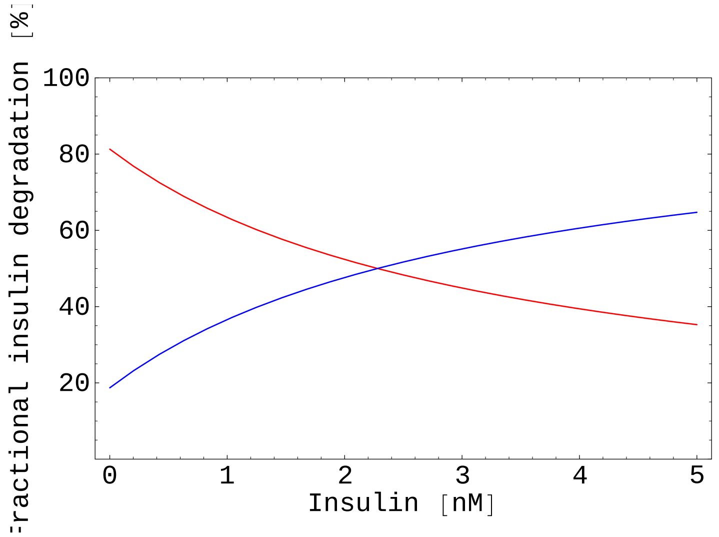


```
- Graphics -

{0.812942}

{0}

{0.763111}

{0.366085}

(* Export["ins_rel_rates_stat.eps",FigFrac,"EPS",
  ConversionOptions→ {"IncludeSpecialFonts"→ True}] *)

(* Output for manuscript: *)

FigsRatesAndFrac =
  GraphicsArray[{FigRates, FigFrac}, ImageSize → 1000, DisplayFunction → Identity];
(* Show[FigsRatesAndFrac,DisplayFunction→$DisplayFunction]; *)
Export["ins_rates_and_frac.eps", FigsRatesAndFrac,
 "EPS", ConversionOptions → {"IncludeSpecialFonts" → True}]

ins_rates_and_frac.eps
```

```
(* Insulin clearance *)

ClearanceLiver = RateLiverFinished * Vplasma * 10^3 * 60 / Ins //. Circulation /. bw → 200;
(* ml/min *)
ClearanceKidney = RateKidney * Vplasma * 10^3 * 60 / Ins //. Circulation /. bw → 200;

ClearanceTotal = ClearanceLiver + ClearanceKidney;

FigClearance = Plot[{ClearanceLiver, ClearanceKidney, ClearanceTotal}, {Ins, 0, 5},
  PlotStyle → {RGBColor[1, 0, 0], RGBColor[0, 0, 1], RGBColor[0, 0, 0]},
  FrameLabel → {"Insulin [nM]", "Insulin clearance [ml/min]"}, Frame → True,
  AxesOrigin → {0, 0}, PlotRange → {All, {0, 11}}, ImageSize → 400]
```

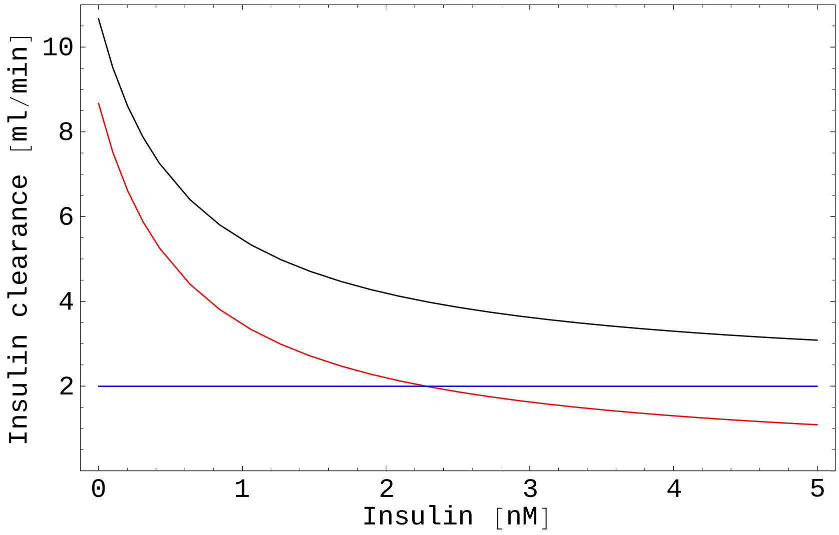


```
- Graphics -

Export["ins_clearance_stat.eps", FigClearance,
 "EPS", ConversionOptions → {"IncludeSpecialFonts" → True}]

ins_clearance_stat.eps

(* Clearance ranges for rats *)

MinInsClearance = Limit[ClearanceLiver, Ins → 0] + ClearanceKidney
MaxInsClearance = Limit[ClearanceLiver, Ins → Infinity] + ClearanceKidney

{10.6686}

{1.99565}
```

```
(* Insulin binding at cell surface *)

<< Graphics`MultipleListPlot`

ReceptorInsBound =
  ((IR + 2 * I2R + IRp + 2 * I2Rp) * Vhep + Insub) / bw /. InsUbstat /. ReceptorBalance /.
    ErgLiver //. Circulation;


(* In silico reproduction of experimental data:
   0.01 nM labeled insulin, varying concentration of unlabeled insulin. *)

ReceptorInsBoundLog = (0.01 / (Ins)) ReceptorInsBound /. Ins → (0.01 + 10^ex);


(* Data points are cell associated labeled insulin [cpm/μg DNA];
 Background (bgr) was substracted *)

Klein2002ins = {{-2, 101.04761904761904`}, {-1.5`, 102.47619047619047`},
   {-1, 100.57142857142857`}, {-0.5`, 79.6190476190476`},
   {0, 47.23809523809524`}, {0.5`, 28.666666666666664`}, {1, 14.857142857142856`},
   {1.5`, 5.333333333333334`}, {2, 0.9523809523809532`}, {4, 0.`}};
Klein2002insEB = {{{-2, 101.04761904761904`},
    ErrorBar[{-3.8095238095238098`, 3.8095238095238098`}]},
   {{-1.5`, 102.47619047619047`}, ErrorBar[
     {-3.8095238095238098`, 3.8095238095238098`}]}, {{-1, 100.57142857142857`},
    ErrorBar[{-3.8095238095238098`, 3.8095238095238098`}]}, {{-0.5`,
     79.6190476190476`}, ErrorBar[{-2.857142857142857`, 2.857142857142857`}]},
   {{0, 47.23809523809524`}, ErrorBar[{-2.857142857142857`, 2.857142857142857`}]},
   {{0.5`, 28.666666666666664`},
    ErrorBar[{-1.9047619047619049`, 1.9047619047619049`}]},
   {{1, 14.857142857142856`}, ErrorBar[{-2.857142857142857`, 2.857142857142857`}]},
   {{1.5`, 5.333333333333334`}, ErrorBar[
     {-1.9047619047619049`, 1.9047619047619049`}]}, {{2, 0.9523809523809532`},
    ErrorBar[{-1.9047619047619049`, 1.9047619047619049`}]},
   {{4, 0.`}, ErrorBar[{-0.9523809523809524`, 0.9523809523809524`}]}};


 (* Projection of model output on experimental data *)

ReceptorInsBoundLogFit = Fit[Klein2002ins, ReceptorInsBoundLog, ex];
FigInsModel =
 Plot[ReceptorInsBoundLogFit, {ex, -3, 4}, PlotStyle → {RGBColor[0, 0, 0]},
  FrameLabel → {"log Insulin [nM]", "Cell associated labeled insulin"},
  Frame → True, DisplayFunction → Identity, PlotRange → {All, All}]


FigInsData = MultipleListPlot[Klein2002insEB,
  SymbolStyle → {RGBColor[1, 0, 0]}, SymbolShape → {PlotSymbol[Box]},
  AxesOrigin → {0, 0}, FrameLabel → {"", "", "", "Cell-associated labeled insulin"},
  Frame → True, DisplayFunction → Identity]
InsData = Show[FigInsModel, FigInsData,
  FrameLabel → {"log insulin [nM]", "Associated labeled insulin [cpm/μg DNA]"},
  DisplayFunction → $DisplayFunction, ImageSize → 400]

- Graphics -

- Graphics -
```

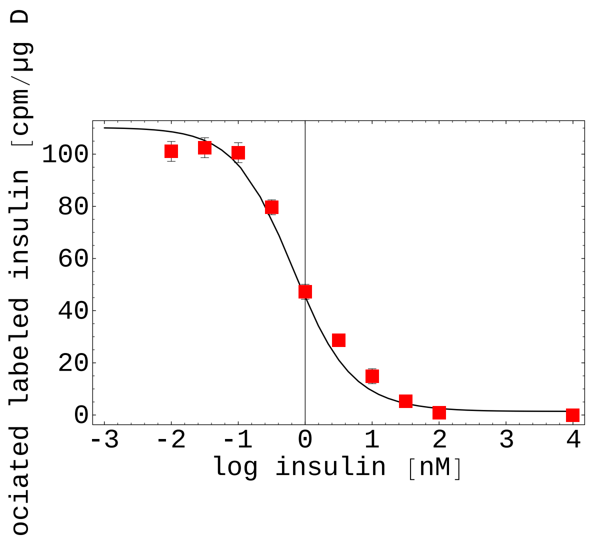
ociated labeled insulin [cpm/μg D
100
80
60
40
20
0
-3
-2
-1
0
1
2
3
4
log insulin [nM]

- Graphics -

```
(* Phosphorylated receptors *)

ReceptorPhosphorylated =
  (Rp + IRp + I2Rp + Rpen + IRpen + I2Rpen) / Rtotal /. ReceptorBalance /. ErgLiver;
ReceptorPhosphorylatedLog = ReceptorPhosphorylated /. Ins → 10^ex;
(*Activity data scaled on fractional receptor phosphorylation*)
(*y-data [cm] extracted from printout of plot *)


Klein2002phos = {{-2, 0.035398230088495575`}, {-1, 0.08495575221238938`},
   {0, 0.39292035398230085`}, {1, 0.6300884955752213`}, {2, 0.6725663716814159`}};

Klein2002phosEB = {{-2, 0.035398230088495575` * facphos},
   {-1, 0.08495575221238938` * facphos}, {{0, 0.39292035398230085` * facphos},
    ErrorBar[{-0.035398230088495575` * facphos, 0.035398230088495575` * facphos}]},
   {{1, 0.6300884955752213` * facphos}, ErrorBar[{-0.07079646017699115` * facphos,
      0.07079646017699115` * facphos}]}, {{2, 0.6725663716814159` * facphos},
    ErrorBar[{-0.08495575221238938` * facphos, 0.08495575221238938` * facphos}]}};

(* Projection of experimental data on model output *)

ReceptorPhosFit = Fit[Klein2002phos, ReceptorPhosphorylatedLog, ex];


FigPhosModel = Plot[ReceptorPhosphorylatedLog, {ex, -2, 2},
   PlotStyle → {RGBColor[0, 0, 0]}, FrameLabel → {"log Insulin [nM]", "Fraction"},
   Frame → True, DisplayFunction → Identity, PlotRange → {All, {0, 0.8}}];

ScalingPhos = ReceptorPhosphorylatedLog / ReceptorPhosFit;


FigAktData =
 MultipleListPlot[Evaluate[Klein2002phosEB /. facphos -> ScalingPhos[[1]]],
  SymbolStyle → {RGBColor[1, 0, 0]}, DisplayFunction → Identity,
  SymbolShape → {PlotSymbol[Box]},
  AxesOrigin → {0, 0}, FrameLabel → {"", "", "", "fmol/(fmol*min)"}, Frame → True]

PhosData = Show[FigPhosModel, FigAktData,
  FrameLabel → {"log insulin [nM]", "Fraction of phosphorylated receptors"},
  PlotRange → {All, All}, DisplayFunction → $DisplayFunction, ImageSize → 400]
```

- Graphics -

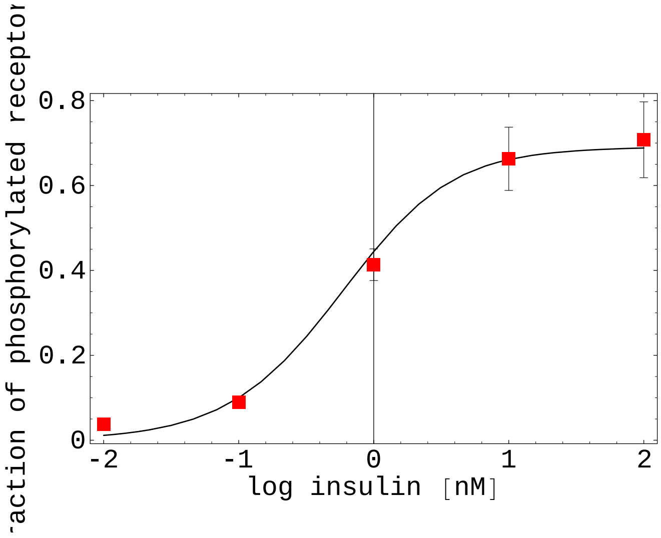

- Graphics -

```
(* Output for manuscript: *)

FigsValidation = GraphicsArray[{InsData, PhosData}, ImageSize → 1000];
(* Show[FigsValidation,DisplayFunction→$DisplayFunction]; *)
Export["ins_validation.eps", FigsValidation,
 "EPS", ConversionOptions → {"IncludeSpecialFonts" → True}]
```

```
ins_validation.eps
```

```
ReceptorIns =
  (Rtotal - R - Rp - Ren - Rpen) / Rtotal /. InsUbstat /. ReceptorBalance /. ErgLiver //.
   Circulation;

ReceptorIns2 =
  (I2R + I2Ren + I2Rp + I2Rpen) / Rtotal /. InsUbstat /. ReceptorBalance /. ErgLiver //.
   Circulation;

ReceptorInsPM =
  (IR + I2R + IRp + I2Rp) / Rtotal /. InsUbstat /. ReceptorBalance /. ErgLiver //.
   Circulation;

ReceptorIns2PM =
  (I2R + I2Rp) / Rtotal /. InsUbstat /. ReceptorBalance /. ErgLiver //. Circulation;
ReceptorInsEN = ReceptorIns - ReceptorInsPM;
ReceptorIns2EN = ReceptorIns2 - ReceptorIns2PM;

(* Percentage of receptors with two bound insulin
  at the basal insulin concentration and at 100 nM insulin *)

ReceptorIns2 * 100 /. Ins → 0.07
ReceptorIns2 * 100 /. Ins → 100
```

```
{0.012203}

{47.8592}
```

```
(* Fraction of receptors with bound insulin (one or two, blue)
  and fraction of receptors with two bound insulin molecules (red) *)

FigInsIns2 = Plot[{ReceptorIns, ReceptorIns2},
  {Ins, 0, 5}, PlotStyle → {RGBColor[0, 0, 1], RGBColor[1, 0, 0]},
  FrameLabel → {"Insulin [nM]", "Fraction occupied receptors"},
  (*RotateLabel→False,*) Frame → True, PlotRange → {All, All}, ImageSize → 400]
FigInsIns22 = Plot[{ReceptorIns, ReceptorIns2}, {Ins, 0, 100},
  PlotStyle → {RGBColor[0, 0, 1], RGBColor[1, 0, 0]},
  FrameLabel → {"Insulin [nM]", "Fraction occupied receptors"},
  (*RotateLabel→False,*) Frame → True, PlotRange → {All, All}, ImageSize → 400]
```

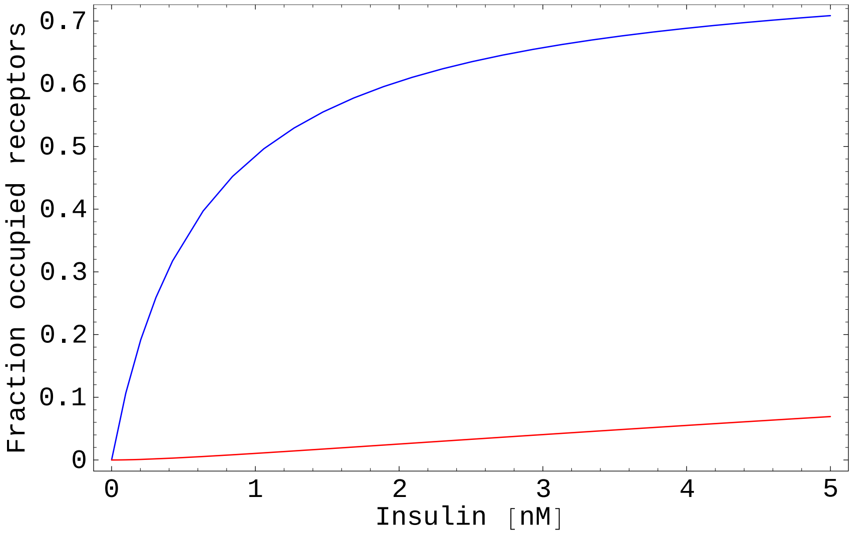

- Graphics -

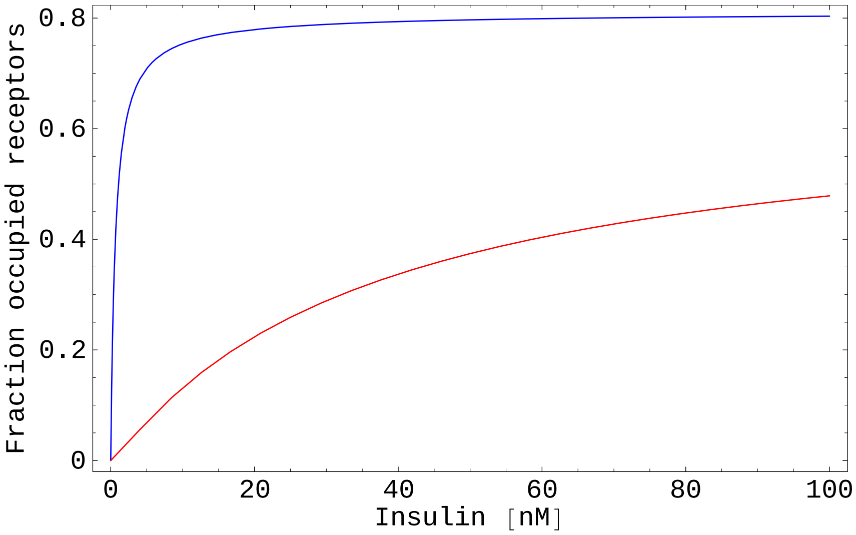

- Graphics -

```
(* Fraction of receptors with 2 insulin molecules over receptors
  with one or two insulin (EN: green, PM: red, total: blue) *)

FigFracInsIns2 = Plot[{ReceptorIns2 / ReceptorIns,
   ReceptorIns2PM / ReceptorInsPM, ReceptorIns2EN / ReceptorInsEN}, {Ins, 0, 5},
  PlotStyle → {RGBColor[0, 0, 1], RGBColor[1, 0, 0], RGBColor[0, 1, 0]},
  FrameLabel → {"Insulin [nM]", "Fraction occupied receptors"},
  (*RotateLabel→False,*) Frame → True, PlotRange → {All, All}, ImageSize → 400]
FigFracInsIns22 = Plot[{ReceptorIns2 / ReceptorIns,
   ReceptorIns2PM / ReceptorInsPM, ReceptorIns2EN / ReceptorInsEN}, {Ins, 0, 100},
  PlotStyle → {RGBColor[0, 0, 1], RGBColor[1, 0, 0], RGBColor[0, 1, 0]},
  FrameLabel → {"Insulin [nM]", "Fraction occupied receptors"},
  (*RotateLabel→False,*) Frame → True, PlotRange → {All, All}, ImageSize → 400]
```

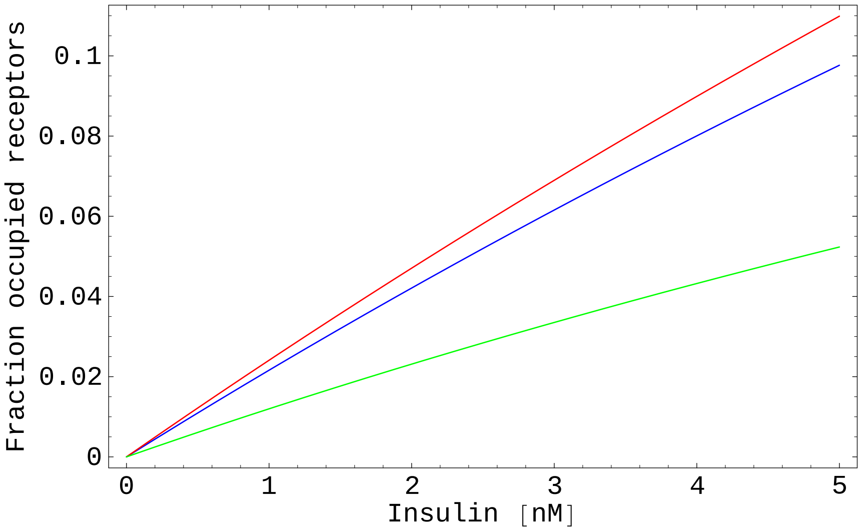

- Graphics -

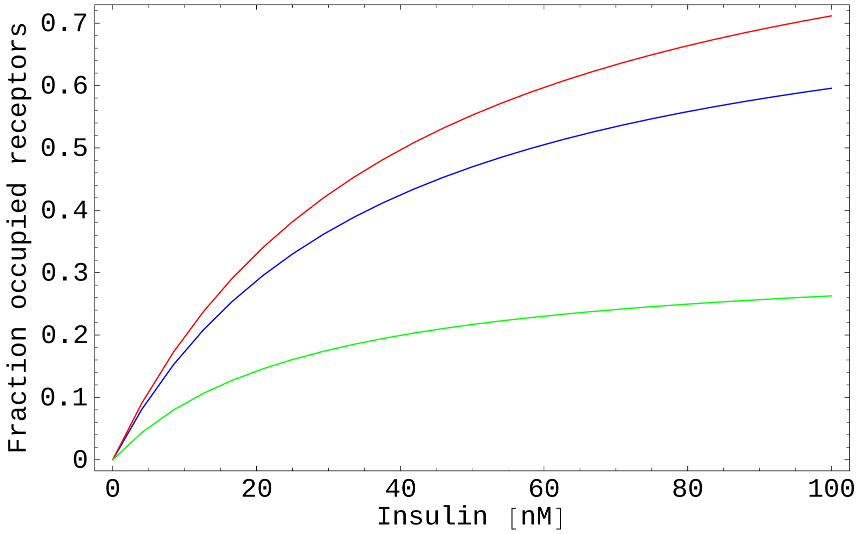

- Graphics -
